# Trade and foreign fishing mediate global marine nutrient supply

**DOI:** 10.1073/pnas.2120817119

**Published:** 2022-05-23

**Authors:** Kirsty L. Nash, M. Aaron MacNeil, Julia L. Blanchard, Philippa J. Cohen, Anna K. Farmery, N. A. J. Graham, Andrew L. Thorne-Lyman, Reg A. Watson, Christina C. Hicks

**Affiliations:** ^a^Centre for Marine Socioecology, University of Tasmania, Hobart, TAS, 7004, Australia;; ^b^Institute for Marine and Antarctic Studies, University of Tasmania, Hobart, TAS, 7004, Australia;; ^c^Ocean Frontier Institute, Department of Biology, Dalhousie University, Halifax, NS, B3H 4R2, Canada;; ^d^WorldFish, Jalan Batu Maung, Batu Maung, Malaysia;; ^e^Australian Research Council Centre of Excellence for Coral Reef Studies, James Cook University, Townsville, QLD, 4811, Australia;; ^f^Australian National Centre for Ocean Resources and Security, University of Wollongong, Wollongong, NSW, 2522, Australia;; ^g^Lancaster Environment Centre, Lancaster University, Lancaster, LA1 4YQ, United Kingdom;; ^h^Center for Human Nutrition, Department of International Health, Johns Hopkins Bloomberg School of Public Health, Baltimore, MD 21205;; ^i^Center for a Livable Future, Department of Environmental Health and Engineering, Johns Hopkins Bloomberg School of Public Health, Baltimore, MD 21205

**Keywords:** micronutrient, seafood, high seas, flag of convenience, aquatic food

## Abstract

Fish are an important source of bioavailable micronutrients and essential fatty acids, and capture fisheries have potential to substantially reduce dietary deficiencies. Vigorous debate has focused on trade and fishing in foreign waters as drivers of inequitable distribution of volume and value of fish, but their impact on nutrient supplies from fish is unknown. We analyze global catch, trade, and nutrient composition data for marine fisheries to quantify distribution patterns among countries with differing prevalence of inadequate nutrient intake. We find foreign fishing relocates 1.5 times more nutrients than international trade in fish. Analysis of nutrient flows among countries of different levels of nutrient intake shows fishing in foreign waters predominantly (but not exclusively) benefits nutrient-secure nations, an outcome amplified by trade. Next, we developed a nutritional vulnerability framework that shows those small island developing states and/or African nations currently benefiting from trade and foreign fishing, and countries with low adaptive capacity, are most vulnerable to future changes in nutrient supplies. Climate change exacerbates vulnerabilities for many nations. Harnessing the potential of global fisheries to address dietary deficiencies will require greater attention to nutrition objectives in fisheries’ licensing deals and trade negotiations.

## Significance

The world produces enough food to nourish the global population, but inequitable distribution of food means many people remain at risk for undernutrition. Attainment of Sustainable Development Goal 2 relies on greater attention to distribution processes that match food qualities with dietary deficiencies. We explore this in the context of fisheries. Foreign fishing and international trade divert nutrients caught in marine fisheries from nutrient-insecure toward nutrient-secure nations. Where nutrient-insecure countries do benefit from foreign fishing and trade, there tends to be high vulnerability to future changes in nutrient flows arising from changes to foreign fishing and trade. This research highlights the need for greater transparency around distribution of fish and for nutrition security to be considered more centrally in development of trade agreements.

Transforming food systems to deliver adequate nutrition to vulnerable populations is a persistent global concern. While an international commitment to end all forms of hunger was first articulated over 75 y ago, poor diets remain the leading cause of mortality and morbidity, with more than three billion people still unable to afford a healthy diet (1, 2). Micronutrient deficiencies are an important, but often hidden, element of malnutrition, driven more by deficient dietary quality than food quantity (*SI Appendix*, Glossary) (3). Micronutrient deficiencies result in substantial economic and health costs to individuals and societies (1, 3), precipitating an estimated one million premature deaths annually, a diversity of physical and cognitive developmental problems, and an estimated reduction in gross domestic product of over 10% for some African nations (2, 4). In many low- and middle-income nations, fish are a vital source of food, particularly where consumption of other animal-based foods is low (Dataset S1, A) (5–7). Fish are an important source of bioavailable micronutrients and essential fatty acids, often providing a greater concentration and diversity of these nutrients than terrestrial animal-source foods (Dataset S1, B) (5, 8, 9). Yet, marine fisheries hold unrealized potential to help address nutrient deficiencies globally; congruence between fisheries, health, and trade policy is now needed to ensure aquatic nutrients are reaching populations vulnerable to undernutrition (7).

Supply of fish to many people, particularly those living further from coasts, is dependent on effective distribution networks. Fisheries’ supply chains can be complex, with price, infrastructure, waste and loss, and formal and informal governance of domestic and international trade mediating the quality, quantity, and ultimate consumers of fish. Prior work has shown for a number of countries with inadequate nutrient intakes that the nutrient potential of catches within their exclusive economic zone (EEZ) exceeds the dietary needs of their coastal populations (<100 km from the coast), suggesting fishery supply chains may be diverting fish from vulnerable populations (7). Foreign fishing fleets and international trade contribute substantially to broad-scale (re)distribution of fish from the site of capture (10–12). Increases in imports or foreign fishing operations may buffer national limits to, or declines in, nutrient availability from wild-capture fisheries (13). However, trade and foreign fishing may also intensify social and economic inequities, and in the case of trade, there is contextual evidence of negative impacts on food and nutrition security (1, 11, 14, 15). Trade is an important structural driver of noncommunicable diseases by increasing availability of low-quality and unhealthy foods (16), and there are increasing concerns that foreign fishing might reduce food security of fished nations (3). Importantly, there is a current lack of understanding of how international trade and foreign fishing—factors known to affect tonnage and value of marine fish supplies (12, 17)—affect the distribution of nutrients. Given that nutrient yields are a function of species composition of the catch (and respective nutrient qualities of those species), rather than simply the combined catch volume (7), novel analyses (over and above catch volume) are required to understand how these distribution processes impact nutrient supplies from fisheries.

Here we use a trait-based model developed by Hicks et al. (7) to estimate the content of seven nutrients (calcium, iron, selenium, zinc, long chain omega-3 polyunsaturated fatty acids [hereafter omega-3], vitamin A, and protein) for marine finfish caught and traded globally. We calculate the reported mass of nutrients caught in the high seas and each EEZ by foreign and domestic fleets and traded among nations. To facilitate comparison of the effects of fishing and trade among nutrients, we express the mass of nutrients in terms of the number of reproductive age females [a demographic where the consequences of nutrient deficiencies can be most extreme (1, 3)] whose recommended nutrient intake (RNI) could theoretically be met by the mass of each nutrient (*Materials and Methods* and *SI Appendix*, Table S1). We show how accounting for flags of convenience (FOCs), where fishing vessel owners reside in a country other than the flagged nation, affects our understanding of who gains the nutritional benefits arising from foreign fishing. Next, we explore flows in nutrients among countries experiencing varying prevalence of inadequate nutrient intake. Finally, combining nutrient availability with exposure to trade and foreign fishing instabilities, we construct a framework to assess future nutrient vulnerabilities, with and without predicted climate change.

## Results and Discussion

### Influence of Foreign Fishing and Trade on Nutrient Supply.

**Fig. 1.**
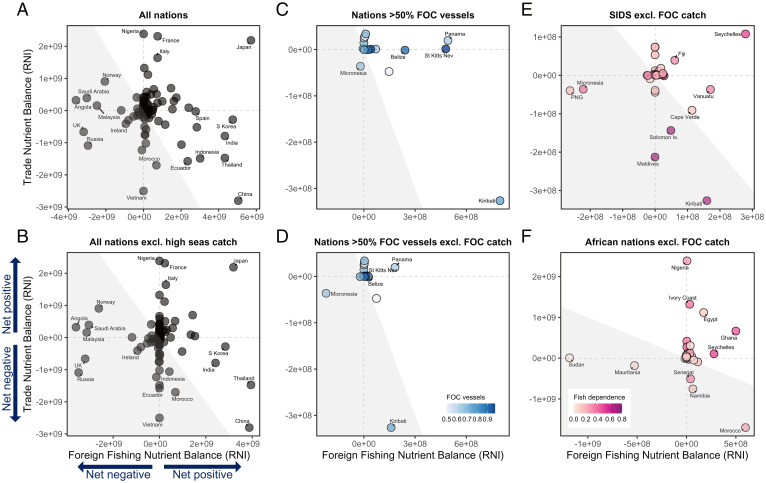
Net gains and losses in fisheries-derived nutrient supply arising from foreign fishing and trade. (*A*) All nations, where foreign fishing balance is number of RNIs provided by fishing in both the high seas and foreign EEZs minus number of RNIs lost to foreign fleets fishing in own EEZ, averaged across nutrients. Trade balance is number of RNIs imported minus number exported. (*B*) All nations, where foreign fishing balance only includes fishing in EEZs. (*C* and *D*) Nations with >50% of fishing vessels under FOCs with and without FOC catches included in foreign fishing balance, respectively. (*E* and *F*) SIDS and African nations, with FOC catches removed from foreign fishing balance. Shading of background in all plots indicates whether combined trade balance and foreign fishing balance is positive (white) or negative (gray). Shading of symbols in *C* and *D* indicates proportion of vessels as FOC, and shading in *E* and *F* indicates the proportion of animal protein consumed that is provided by fish, an indicator of dependence on fish for food. RNIs are based on adult females (*SI Appendix*, Table S1).

Over 1.5 times the amount of fishery-derived nutrients are moved through foreign fishing compared with international trade, suggesting foreign fishing is the dominant process redistributing these nutrients. Many nations (64%; *SI Appendix*, Table S2A) benefit by harvesting fish from foreign waters, but several countries benefit disproportionately, including Japan, South Korea, and China (Fig. 1*A*). In some instances (e.g., Ecuador and Indonesia), the positive foreign fishing balance is reliant on catches from the high seas rather than from EEZs of other countries (Fig. 1*A* vs. Fig. 1*B*). When reported FOCs are accounted for in the foreign fishing balance, the percentage of countries benefiting from foreign fishing remains at 64% (*SI Appendix*, Table S2A). However, the magnitude of those benefits drops for nations where high percentages of their flagged fishing vessels have owners residing in other nations (Fig. 1*C* vs. Fig. 1*D*). For example, the foreign fishing balance of Kiribati drops by 80% when FOC catches are removed. Many of the nations with high percentages of FOCs within their fleets are small island developing states (SIDS) and/or African nations, (e.g., Sierra Leone, St. Kitts and Nevis, and Kiribati). These countries experience nutrient insecurity and are highly reliant on fish for food (mean prevalence of inadequate nutrient intake of 19% and >20% of animal protein from fish, for countries with ≥50% FOC vessels) (18, 19). Over 30% of nations experience net nutrient losses from foreign fishing, increasing to 60% when the high seas are excluded, with Norway, Angola, Saudi Arabia, Malaysia, and the United Kingdom experiencing the greatest losses (Fig. 1 *A* and *B*). Arguably, these flows can only be considered losses if the source nation has the capacity to harvest what foreign vessels are currently taking from their waters (current loss) or where current foreign catches negatively impact the future status of local stocks (loss of potential).

We found 63% of countries receive net gains in nutrients through international trade, with Nigeria, France, Japan, and Italy experiencing the greatest benefits. These benefits are largely supported by a few prominent exporters, including China and Russia, who export a greater quantity of nutrients from fish, as well as tonnage and value (20), than they import (Fig. 1*A*). In contrast, 36% of nations have net trade losses, of which over 50% are SIDS and/or African nations (Fig. 1 *E* and *F*).

Collectively, nearly half of nations experience net gains in nutrients from fish through access to both imports and foreign fishing (upper right quadrant in Fig. 1 and *SI Appendix*, Table S2B), and nearly three quarters of nations experience net gains from fish when imports and foreign fishing are summed (white background shading in Fig. 1 and *SI Appendix*, Table S2B) with Japan receiving the greatest of such benefits. Few (11%) nations experience net losses in nutrients from both foreign fishing and trade (bottom left quadrant in Fig. 1 and *SI Appendix*, Table S2B), and 28% of nations experience net losses when foreign fishing and trade are summed (gray background shading Fig. 1 and *SI Appendix*, Table S2B). The United Kingdom experiences one of the greatest net losses, but recent policy changes in the UK Fisheries Act (2020) look to give full national control over foreign fishing access (21), potentially reducing nutrients losses in the future. Half of the nations that experience net losses from both foreign fishing and trade are SIDS and/or African nations, with high levels of fish consumption and nutrition insecurity (Fig. 1 *E* and *F*).

These fishing and trade balances need to be considered in relation to the realities of fishing patterns and fish distribution networks. Foreign fleets may unload catch in local ports for subsequent export or may transship catch from multiple fishing vessels to large, refrigerated carrier ships (22, 23). Furthermore, FOCs are often opaque and have been associated with illegal and unreported activities (24). For example, only 1% of Ghana’s fleet are reported as FOCs (19), and yet over 90% of her trawlers are estimated to have some degree of Chinese ownership (24). Similarly, tracking trade of seafood products is complicated by circular trade flows—where nations import raw product and then reexport in processed form (10, 25). For example, Norway is a major seafood producer and exporter; however, Fig. 1 shows Norway as a net importer of fisheries-derived nutrients, mainly from China. A large part of this inconsistency is due to the dominance of salmon aquaculture in Norwegian seafood exports, fish which are not included in our wild capture trade analyses. However, an additional consideration is the likelihood that Norway is shipping fish to China to benefit from cheaper processing labor and then reimporting the processed form but only reporting the import step. Such discrepancies have been reported for other nations, such as the United States, with the potential consequence of misinformed policy decisions (25). These complexities mean that the national gains and losses we estimated in relation to foreign fishing and trade show reported nutrient supplies globally but, in some instances, may not equate to the supply of nutrients available in the flagged country. Product traceability improvements would illuminate these patterns and their consequences on the nutrition potential of fisheries.

A final consideration when viewing our findings is the presence of shared or disputed EEZs. The EEZ areas we used in the catch dataset are from https://www.marineregions.org/eez.php, where EEZs are projected for each country based on provisions of the United Nations Convention on the Law of the Sea. In some instances, catch may be classified as foreign fishing when the EEZ is in fact contested or overlapping. This may lead to more negative foreign fishing balances than might be expected, e.g., Saudi Arabia (Fig. 1*A*) and Sudan (Fig. 1*F*).

### Distribution as a Driver of Food and Nutrition Security.

To understand how foreign fishing and trade of fishery-derived nutrients may support or undermine nutrition security, we estimated flows (mass) of nutrients among nations with differing prevalence of inadequate nutrient intake (*Materials and Methods*) for four micronutrients that are known to be relatively concentrated and bioavailable in fish and where national intake data are available (calcium, iron, zinc, and vitamin A) (18). We express these flows in terms of changes in nutrient yields (percentage of RNI per capita, where RNI is for reproductive aged females). The potential impact of fishery-derived nutrients on nutrition security will be impacted by the importance of fish in local diets. Thus, we estimated nutrient flows and yields in two ways, first accounting for all nations’ level of inadequate micronutrient intake (*SI Appendix*, Table S3A) and second focusing on those nations which were highly dependent on fish (>10% of animal sourced protein from fish). Furthermore, understanding how distribution of fishery-derived nutrients may affect nutrition security is complicated by FOCs and transshipment. To understand the impact of FOCs, we also estimated nutrient flows and yields accounting for nations issuing FOCs.

**Fig. 2.**
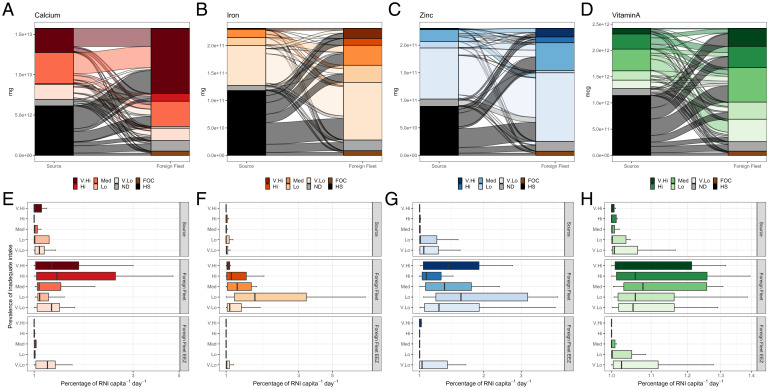
Annual flow and yield of fishery-derived nutrients due to foreign fishing accounting for FOCs. (*A*–*D*) Flow of calcium (mg), iron (mg), zinc (mg), and vitamin A (mcg) due to foreign fishing for nations grouped by prevalence of inadequate intake of respective nutrients in source countries and foreign fleet (sink) countries. (*E*–*H*) (*Top*) Yields (log10 percentage of RNI capita^−1^ d^−1^) extracted from source nation’s EEZs, grouped by source nations’ nutrient intake. (*Middle*) Yields caught by foreign fleets in EEZs and the high seas, grouped by foreign fleet (sink) nations’ nutrient intake. (*Bottom*) Only catches from foreign EEZs. Prevalence of inadequate nutrient intake categories (*SI Appendix*, Table S3A): V.Hi (dark shading), countries with >50% of population with inadequate intake of respective nutrient; Hi, 25 < % ≤ 50; Med,10 < % ≤ 25; Lo, 5 < % ≤ 10; V.Lo (light shading), ≤5%; ND (gray shading), no deficiency data available for these nations; FOC (brown shading), flows to FOCs; HS (black shading), nutrients sourced from high seas.

By sourcing considerable quantities of nutrients from the high seas, foreign fishing appears to drive an increase in nutrients across most categories of inadequate intake (Fig. 2 *A*–*D*). The impact of these flows on per capita nutrient yields, and thus potential nutrient intake, varies among nutrients (Fig. 2 *E*–*H*, *Top* and *Middle*). For example, the greatest increases in apparent per capita supply of calcium from foreign fishing occur in countries with high and very high levels of inadequate calcium intake (dark shading; ∼4,000- and ∼750-fold increase in % RNI per capita for these respective intake categories). This finding may be influenced by the widespread prevalence of high and very high inadequate calcium intake (*SI Appendix*, Table S3B) (18) but suggests that high seas fishing may be an important provider of calcium supplies in nutrient-deficient contexts. However, our country-level analysis is unable to determine if these supplies reach the most nutritionally vulnerable sectors of society: domestic markets, price, preference, waste and loss, and intrahousehold dynamics will mediate that (e.g., ref. 26). For example, species sourced from the high seas tend to be expensive (27), which means these landings are more likely to be consumed by wealthier demographics within these nations. In contrast to calcium, the greatest increases in apparent supply of iron due to foreign fishing occur in countries with low levels of inadequate iron intake (light shading; 20-fold increase in % RNI per capita). Japan represents such a country with low levels of inadequate iron intake—the daily recommended intake of iron of nearly two million Japanese could be met by the fish caught by their foreign fishing fleet.

Increases in nutrient yields per capita from foreign fishing in EEZs (i.e., when excluding high seas catches) are more moderate (Fig. 2 *E*–*H*, *Bottom*) and predominantly benefit countries with low prevalence of inadequate nutrient intake (light shading). This is not surprising given the small number of high-income countries that dominate fishing in foreign EEZs (11), experience low prevalence of inadequate nutrient intake [7.4% vs. global mean of 17% (18)], and tend to focus fishing effort around low- and middle-income nations (11). For example, the greatest yields from fishing in other nations’ EEZs are caught by Iceland for all four nutrients (7 to 44% of RNI per capita). Iceland is not recognized as one of the dominant foreign fishing nations (Fig. 1) (11), rather the large yields can be explained by the relatively small population size. These findings add to concerns about agreements to fish in low- and middle-income nations (11, 28, 29), highlighting that such agreements reduce nations’ ability to direct their own food production toward citizens experiencing nutritional insecurity.

These overall trends hold across the different scenarios analyzed, i.e., where the relative importance of fish to local diet is accounted for or not (*SI Appendix*, Fig. S1 vs. Fig. 2), and whether FOCs are considered in the analysis or not (Fig. 2 vs. *SI Appendix*, Fig. S2). However, our findings may underestimate the scale of inequity in nutrient flows arising from FOCs. Our analysis accounted for reduced flows of nutrients to countries issuing FOCs: countries that are often nutrient insecure and reliant on fish as the dominant animal-sourced food (18, 19). However, we were unable to account for the true nationality of FOC vessels and thus the nationality catching these “lost” nutrients; i.e., we cannot track where these nutrients ended up. Nonetheless, records of FOC ownership show over 60% of FOC vessels have owners living in just 10 nations (23). These are countries where nutrient deficiencies are generally low and the population is less reliant on fish (mean prevalence of inadequate nutrient intake 10% and 11% of animal protein from fish for top 10 nations using FOCs) (18, 23), suggesting FOC tagged nutrients (brown shading in Fig. 2 *A–D*) are likely flowing to areas with populations experiencing lower nutritional need. Few data are available on transshipment practices, but there is a strong correlation between nationalities issuing FOCs and transshipment practices (30), such that even more nutrients may be diverted from nations providing flags than our analysis suggests. Greater transparency in vessel flagging and transshipment practices would help reduce these uncertainties and inform action to guide reduction in inequalities in the distribution of fishery-derived nutrients.

Trade also impacts nutrient flows among nations, although the magnitude of these effects is smaller than for foreign fishing (*SI Appendix*, Fig. S3 *A–D*). Trade tends to drive flows of nutrients away from countries experiencing high and very high prevalence of inadequate nutrient intake (dark shading), toward countries experiencing low levels of inadequate nutrient intake (light shading). Due to the greater number of countries (and their larger populations) importing versus exporting fish, a decline in the after-trade nutrient yield was observed across most categories of inadequate micronutrient intake (very low to very high; *SI Appendix*, Fig. S3 *E–H*). Nonetheless, after-trade losses were greater for countries experiencing greater prevalence of inadequate nutrient intake than countries with low prevalence. Once again, these overall trends hold whether the relative importance of fish to local diet is accounted for or not (*SI Appendix*, Fig. S3 vs. Fig. S4).

Research into the dollar value of fish traded among nations has shown wealthy nations tend to buy high-value fish products and sell low-value products to poorer nations (12, 17). Yet, when we examined the distribution of nutrients, we found no substantive correlations (Spearman rank less than ±0.27) between the net foreign fishing or trade balance for each nutrient and three metrics of national socioeconomic status—coefficient of human inequality, gross domestic product (GDP) per capita, and human development index (see *SI Appendix*, Fig. S5, for full details). This suggests, at a global scale, redistribution of nutrients from fisheries does not follow consistent pathways of buying power. However, the shifts in per capita nutrient yields (Fig. 2 and *SI Appendix*, Figs. S1–S4) suggest that when nutritional differences among fish species and nations’ nutritional needs are considered, distribution of fish supplies may be undermining nutrition security and equity (15–17, 31).

### Vulnerability of Nutrient Supply.

The global food system is increasingly interconnected. These connections influence how social, economic, and environmental changes play out, affecting food and nutrition security (32). International connections that support current net nutrient gains may mask vulnerabilities to geographically distant shifts in fisheries and trade policy. These spatially distant changes may propagate as shocks through the food system (32, 33). There is compelling evidence that size and frequency of food shocks are increasing (33, 34), and the COVID-19 pandemic illustrates the significant impact of a large-scale shock on production and global supply chains (35–37). Thus, if society is to maximize the role of fishery-derived nutrients in nutrition security, current gains and losses in nutrient supply arising from foreign fishing and trade must be considered alongside future vulnerabilities in supply.

To assess nations’ vulnerability to changes in fisheries-derived nutrient supply, we adapted a commonly used vulnerability framework (38) (*SI Appendix*, Fig. S6):

Vulnerability=Exposure+Sensitivity – Adaptive Capacity.

**Fig. 3.**
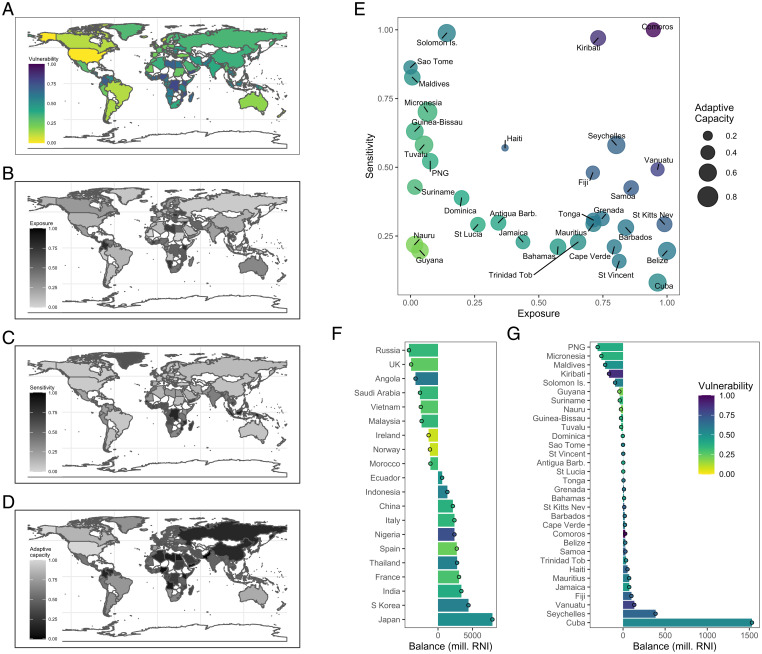
National vulnerability to changes in nutrient supply due to foreign fishing and trade. (*A*) Map of vulnerability to changes in fisheries-derived nutrients. Vulnerability calculated from (*B*) exposure to changes in trade and foreign fishing plus (*C*) sensitivity to loss of fisheries-derived nutrients minus (*D*) adaptive capacity to changes in nutrient supplies. (*E*) Exposure, sensitivity, adaptive capacity, and vulnerability of SIDS. (*F* and *G*) Vulnerability of countries in relation to their 2015 trade and foreign fishing balance, showing countries with greatest gains and losses (highlighted in Fig. 1*A*) and SIDS (highlighted in Fig. 1*E*), respectively. Nutrient balance is measured in millions of RNIs and sums trade and foreign fishing balances (Fig. 1).

We included metrics of exposure to changes in nutrient supply from foreign fishing and trade based on volatility and redundancy in these supplies, sensitivity to a loss of fishery-derived nutrients arising from reliance on fish for food and prevalence of inadequate nutrient intake, and capacity to adapt to this loss based on national economic, health, and trade-based factors (*Materials and Methods* and *SI Appendix*, Table S4). The countries exhibiting the greatest vulnerability were primarily in Africa and Pacific SIDS (Fig. 3 *A* and *E*), where populations are also experiencing the greatest growth in need for nutrients (39). The mechanism driving vulnerability varied among regions: for example, exposure was highly variable among African nations (Fig. 3*B*) and was driven primarily by changes in imports (*SI Appendix*, Fig. S7*A* vs. Fig. S7*B*). Sensitivity was also variable across Africa and Pacific SIDS (Fig. 3 *C* and *E*), primarily driven by differences in dietary dependence on fish (*SI Appendix*, Fig. S7*C* vs. Fig. S7*D*), while adaptive capacity tended to be low in Africa and was variable for Pacific SIDS (Fig. 3 *D* and *E*). Critically, within regions, those countries that currently experience nutrient gains from trade and foreign fishing tend to have greater vulnerability to future changes in nutrient supplies than countries currently experiencing nutrient losses (Fig. 3 *F* and *G*). There are, however, exceptions such as Kiribati and Angola, which showed a net loss in nutrients when flows due to trade and foreign fishing were summed but also appear to show high vulnerability to changes in nutrient supplies in the future (Fig. 3*G*).

**Fig. 4.**
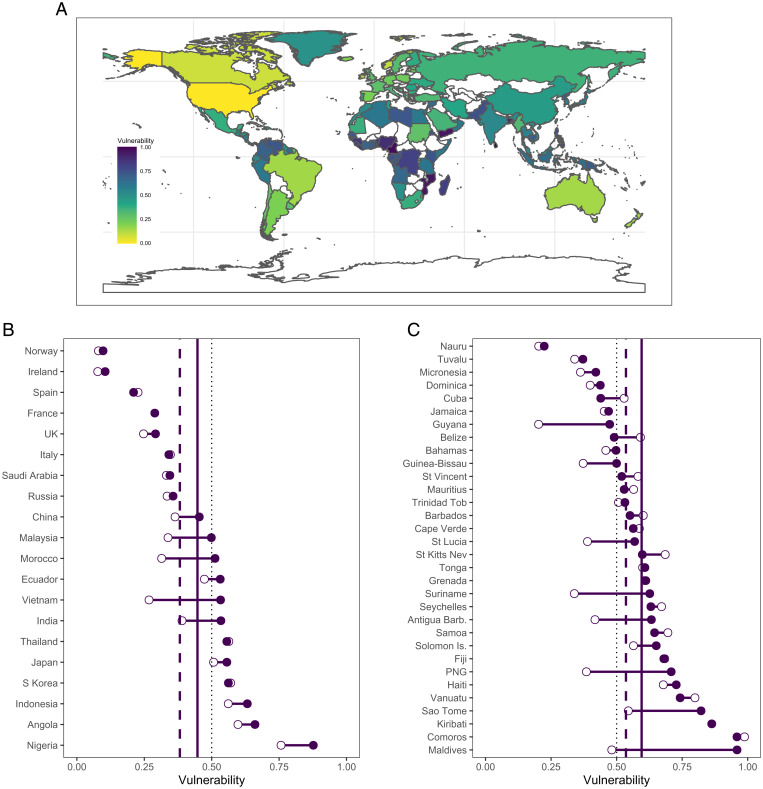
National vulnerability to changes in nutrient supply due to changes in foreign fishing, trade, and climate. (*A*) Map of vulnerability to changes in supply of fisheries-derived nutrients. Difference between vulnerability due to distribution processes (foreign fishing and trade; empty circles) and distribution processes and climate change (filled circles) (*B*) for countries with greatest 2015 gains and losses in nutrients and (*C*) for SIDS. Dotted line, vulnerability of 0.5; purple dashed line, mean vulnerability due to distribution processes for nations included in plot; solid purple line, mean vulnerability due to distribution and climate change.

Trade and foreign fishing are occurring against a backdrop of climate change that is modifying fish communities and driving range shifts and is predicted to lead to overall declines in fisheries production (40, 41). To evaluate the potential compounding effects of climate on distribution-driven vulnerabilities, we integrated our vulnerability framework with information on exposure to climate-driven changes in domestic catches (*Materials and Methods* and *SI Appendix*, Figs. S8 and S9). The addition of projected climate change exacerbated the nutrient vulnerability of many nations (Fig. 4), with the greatest impacts among tropical countries and SIDS. As a result, even SIDS such as Papua New Guinea and Guyana, which experienced relatively low vulnerability in relation to trade and foreign fishing, show a substantial increase in vulnerability with the addition of exposure of domestic fisheries to climate change (Fig. 4*C*).

### Addressing Nutrient Vulnerabilities.

Nutrition insecurity is a pressing global concern, and fisheries have the potential to reduce the burden of malnourishment (7). We have shown that foreign fishing and international trade exacerbate nutrition insecurity, moving fish away from source nations experiencing high prevalence of inadequate nutrient intake. If nations are to address the burden of malnutrition, decision-makers must consider nutrients derived from fisheries as a key resource that needs protection and can be mobilized to address nutrition needs, particularly for more vulnerable sectors of society. Adjustments to trade policy, fishing agreements, environmental management, and public health investments and within nation distribution processes are necessary to improve nutrition outcomes (1, 3, 42).

Food industry and government revenue generation strongly influence negotiations around trade and fishing agreements, often at the expense of public health interests (15, 16). The disparity between binding international trade agreements and nonbinding fisheries management, climate change, and food security agreements (e.g., ref. 43) creates a power imbalance whereby multinational companies have legal capacity to oppose national policies focused on improving fisheries sustainability and public health if those policies are shown to be barriers to trade (15, 44). To counter these imbalances, it is important that food and nutrition security actors (including people working in food, agriculture, and health sectors) are included in trade and foreign fishing negotiations, ensuring that when trade agreements are developed, industry concerns are not prioritized at the expense of nutritional outcomes (45, 46).

Vulnerability to future changes in fishery-derived nutrient supplies is most extreme in those nations with net gains in nutrients from foreign fishing and trade and high reliance on fish for food (e.g., SIDS and African nations). Developing risk-prepared policies relies on understanding the specific mechanisms underlying a nation’s nutritional vulnerability (Figs. 3 and 4). For example, high exposure to changes in trade and foreign fishing may be countered by strengthening domestic nutrient production and distribution systems. In contrast, high sensitivity suggests a need for a diversity of programs aimed at reducing national levels of nutrient deficiencies.

Finally, supporting food and nutrition security actors in improving nutritional outcomes and reducing nutrient vulnerabilities will be enabled by 1) improved understanding of nutrient flows through illegal, unreported, and unregulated fishing; 2) illuminating the role of legitimate informal sectors; and 3) increased transparency in relation to vessel ownership (FOCs) (23), transshipment practices (11), and circular trade flows (25). This would move us to a more complete picture of on-the-ground supplies of fishery-derived nutrients and thus their potential to support future nutrition security.

## Materials and Methods

### Catch and Trade Data.

We sourced spatially explicit wild caught marine fisheries data from the catch reconstruction database of Watson and Tidd (47) (see *SI Appendix*, Supplementary Methods and Table S5, for more details). We chose this catch database because it was closely matched to the only available global seafood trade database (described below in this section) where broad traded commodities are translated to useful taxonomic groupings required for nutrient calculations. This allowed the comparison of landings and seafood trade between countries.

The catch dataset provides tonnage of reported landings at a range of taxonomic resolutions, from species up to broad classes such as “marine finfishes.” Landings are classified according to the source EEZ or high seas, and the nationality of the fishing fleet, allowing for differentiation between fish caught by domestic fishers and foreign fishing vessels. Nationality here and throughout the analyses refers to different countries, states, and territories. Landings data from 2015 were used for the analysis of catch of nutrients. Data from 1976 to 2015 were used to assess variability in the tonnage of fish caught by foreign fishing vessels over time (see vulnerability framework).

We sourced international trade data from Watson et al. (10) (*SI Appendix*, Supplementary Methods and Table S5). These data uniquely report trade of taxa at a range of taxonomic resolutions (rather than commodities), from species up to broad classifications such as marine finfishes. Trade flows are identified according to importing and exporting country. International trade data for 2015 were used for the analysis of trade in nutrients. Data from 1976 to 2015 were used to assess variability in the tonnage of fish traded over time (see vulnerability framework).

These catch and trade data provide an invaluable record for understanding distribution of reported fish products. However, some uncertainties remain, for example, use of FOCs, whereby the beneficial owner of a vessel resides in a different country to the flagged nationality of the vessel. FOCs are attractive to vessel owners as they allow access to the flagged waters, they can be used to mask illegal practices, and rules and regulations may not be as strict or as strictly enforced in host countries (19, 24). We use reported landings in our analyses; however, it is not clear if these FOC-derived nutrients are available to the local population in the issuing country or benefit the country of the beneficial owner, e.g., through shipment. Often the issuing countries are low- and middle-income countries with high prevalence of inadequate nutrient intake (e.g., Kiribati) (19), and thus, these nutrients may not be supporting nutrition of the vulnerable populations that the catch reconstruction data indicate. As a result, we explored patterns of nutrient flows in two ways, first, based on nutrients flowing away from nations providing FOCs and second, based on nutrient flows classified according to the original reported data. To control for FOCs we used a database detailing the proportion of each nations’ vessels under FOCs from Petrossian et al. (19) (*SI Appendix*, Table S5). We multiplied catches for each nation by the proportion of vessels under FOC (averaged across 2013 and 2018). This is a coarse measure of the impact of FOCs on nutrient supplies as 1) the FOC database only included vessels >100 gross tons (19) and 2) we were unable to account for variability in the tonnage or taxonomic identity of fish caught among FOC and non-FOC vessels on a national register. Nonetheless, we considered this adjustment to be a reasonable estimate of the influence of FOCs on nutrient flows as we were only using reported catch data—the majority of which is catch from industrial fishing and thus likely to correspond with the vessels included in the FOC database.

### Traits and Predicting Nutrient Content.

We estimated the nutrient concentration of all taxa reported at family, genus, or species level, which were caught and traded globally in 2015, using the trait-based Bayesian hierarchical model developed by Hicks et al. (7) (*SI Appendix*, Table S5). This model predicts the concentration of seven nutrients that are essential to human health and are bioavailable in fish species (protein, iron, calcium, zinc, selenium, long chain polyunsaturated omega-3 polyunsaturated fatty acids, and vitamin A) from a series of traits linked to the diet (feeding pathway [pelagic or benthic] and trophic level), energy demand (maximum length, age at maturity, K, and body shape), thermal regime (maximum depth and geographical zone), and habitat of species.

We sourced trait information from FishBase (48) (*SI Appendix*, Table S5) for all fish species identified in the catch and trade data. Where trait data were missing for a particular species, genus-level averages were calculated (mean for continuous traits and mode for categorical traits). Where genus-level averages were not available owing to missing data, family-level average values were calculated.

All the analyses focused solely on finfish taxa because 1) the majority of landed catch are finfish (49) (in 2015 these represented 100% of the reported catch in 59 of EEZs, more than 90% of the catch in 141 EEZs, 75% of the catch in 194 EEZs, and 69% of catch from the high seas) and 2) it was not possible to model the nutrient concentrations for invertebrate species, as there are currently insufficient trait data available for these taxa to allow estimation using the trait-based model (7, 49).

### Mass of Nutrients Caught and Traded.

We explored the nutrient content of fish in relation to seven nutrients that are important for human health and for which fish are an important, bioavailable source (50, 51). The hierarchical model (7) provided expected nutrient concentrations (per 100 g raw, edible portions) for all taxa reported at species, genus, and family levels in the catch and trade data. The mean of the posterior probability distributions from the hierarchical model was used for these nutrient concentrations. The mass of nutrients caught and traded was then calculated using the tonnage multiplied by the nutrient concentration for each of the seven nutrients. Mass of nutrients was estimated for the catch from each EEZ (separated by domestic and foreign fishing) and from the high seas. Similarly, we estimated the mass of nutrients traded from each exporting country to each importing country.

Where catch and trade information were provided at taxonomic resolutions below family and therefore nutrient concentrations were not available from the hierarchical model, the mass of nutrients was estimated using a four-tier interpolation approach (*SI Appendix*, Supplementary Methods).

It should be noted that nutrient supplies estimated here do not directly equate to nutrient biomass available for consumption, due to different ways of processing, preparing, and consuming fish that result in variability in the edible biomass (52). We did not convert total biomass to edible weight due to lack of conversion factors for many species, significant inconsistencies in these conversion factors where they are available, and variability in what is considered an edible portion (depends on how the fish is prepared and varies across countries and fish species) (53, 54). Future work directed at quantifying conversion factors for a wider range of fish species and traded commodities is needed.

Our analyses rely on reported landings and as such do not explore flows of nutrients arising from illegal and unreported catches. We focused on reported landings due to the difficulties associated with assigning taxonomic identity (to infer nutrient content) (49) to catches that have not been reported and to ensure the catch data we used were consistent with the trade dataset which was informed by reported catches. Increased transparency in the distribution of global fisheries catches and trade in fish products is needed to allow more comprehensive treatment of flows of fishery-derived nutrients and uncover potential inequalities in these flows. Similarly, we do not account for discards. While discarded fish are not currently available for consumption, future research to explore the scale of potential nutrient production from discards would be useful in contexts of high deficiency risk, where channeling these nutrients to human consumption may be beneficial for nutrition security (55).

### Influence of Foreign Fishing and Trade on Nutrient Supply.

To facilitate comparison of the effects of fishing and trade among the seven nutrients, we expressed the mass of nutrients in terms of the number of reproductive age females [a demographic where the consequences of nutrient deficiencies can be most extreme (1, 3)] whose RNI could theoretically be met by the mass of each nutrient (*SI Appendix*, Table S1).

To estimate the impact of trade and foreign fishing on a nation’s fishery-derived nutrient supply, we calculated the balance between gains and losses arising from either foreign fishing (FFB) or trade (TB) as follows:

[1] $$\mathrm{FFB}i_{n}=\;FFi_{n}–\;\mathrm{LossFF}i_{n},$$

where *FFi_n_* is the number of RNI of nutrient *n* caught by nation *i* fishing in other countries’ EEZs or in the high seas and *LossFFi_n_* is the number of RNI of nutrient *n* caught by other countries’ foreign fleets in nation *i*’s waters. Positive values indicate that nation *i* gains more of nutrient *n* from fishing in foreign waters and the high seas than is extracted by other nations’ fleets fishing in *i*’s EEZ.

[2] $$TBi_{n}=\;\mathrm{IMP}i_{n}–\;\mathrm{EXP}i_{n},$$

where *IMPi_n_* is the number of RNI of nutrient *n* imported by nation *i* and *EXPi_n_* is the number of RNI of nutrient *n* exported to other nations by nation *i*. Positive values indicate that nation *i* gains more RNI of nutrient *n* from imports than it exports, that is, the country is a net importer for nutrient *n*.

Mean foreign fishing balance (averaged across nutrients) was plotted against mean trade balance, to identify which countries gain nutrients from both foreign fishing and trade and which countries experience a loss in nutrient supply due to foreign fishing and/or trade arrangements. For countries that issue FOCs, where the beneficial owner lives in a different country to the flagged nation, we estimated foreign fishing balance both accounting for and not accounting for losses due to FOCs, to understand the potential impact of FOCs on nutrient supplies.

### Distribution as a Driver of Food and Nutrition Security.

To evaluate how the redistribution (via foreign fishing and trade) of fishery-derived nutrients may support or undermine nutrient security, we explored the flow of nutrients due to foreign fishing and trade among countries experiencing different levels of inadequate nutrient intake for the four micronutrients where intake data were available: calcium, iron, zinc, and vitamin A (18) (*SI Appendix*, Table S5). Countries were categorized as having very low to very high prevalence of inadequate intake for each nutrient (*SI Appendix*, Table S3A) according to their status in 2011. In this context, inadequate nutrient intake is based on 1) apparent consumption of all foods (marine, terrestrial, and freshwater) not just wild capture fisheries and 2) the estimated average micronutrient requirements of different demographics accounting for the bioavailability of nutrients in different food sources (see ref. 18 for full details). Sankey diagrams of these nutrient flows were plotted using the ggalluvial package in R (56), and source and sink countries were grouped according to their prevalence of inadequate intake of the respective nutrients. These plots show how flows of fishery-derived nutrients are moving between countries with different levels of nutrient insecurity, i.e., how fisheries trade and foreign fishing may be exacerbating or reducing nutrient insecurity. We ran this analysis in three ways: for all nations, for those highly dependent on fish (>10% of animal sourced protein from fish), and accounting for FOCs.

To understand how this flow of nutrients had the potential to change per capita level supplies of nutrients, the national yield (percentage of RNI per capita per day) of nutrients were calculated within the different nutrient intake categories. Specifically, the median yield (percentage of RNI per capita per day) of each nutrient was estimated in relation to 1) foreign fishing catches for the source nation (source) and the nation that owned the foreign fishing fleet (sink foreign fleet) including and excluding high seas catches and 2) trade for the exporting nation and the importing nation. These data were plotted using a log10(x + 1) transformation.

To understand if there are correlations between socioeconomic status of a nation and patterns in the redistribution of fishery-derived nutrients due to trade and foreign fishing, we ran Spearman rank correlations between the trade and foreign fishing balance of each nutrient and three metrics of national socioeconomic status—coefficient of human inequality, GDP per capita, and human development index (*SI Appendix*, Table S5).

### Vulnerability of Nutrient Supply to Changes in Foreign Fishing and Trade.

We explored vulnerabilities in the supply of nutrients associated with changes in foreign fishing and trade by adapting a well-recognized climate vulnerability framework outlined by ref. 38 and used in the context of fisheries by refs. 6 and 33, among others:

[3] Vulnerability = Exposure + Sensitivity – Adaptive Capacity,

where the vulnerability of a nation’s fisheries-derived nutrient supply is dependent on national 1) exposure to changes in foreign fishing and imports, 2) intrinsic sensitivity to these changes, and 3) capacity to adapt to these changes.

An overview of our vulnerability framework and calculation and scaling of constituent variables are provided in *SI Appendix*, Fig. S6 and Table S4. Note that we used the foreign fishing data that accounted for FOCs in these analyses. We focused on imports rather than trade in general for two reasons: First, we were interested in direct effects on fish-derived nutrient supply and assumed that a loss of fish imports would have direct negative impacts on national supply of fish-derived nutrients, whereas a decline in exports would increase retention of these nutrients in the nation, supporting nutrient supply. We acknowledge that changes in trade will affect overall nutrient supply. For example, a reduction in exports and loss of the revenues they provide will affect a nation’s capacity to buy other nutrient-rich foods—this may affect food security but was not accounted for by our framework. Second, prior work has shown the central role imports play in driving vulnerability to shocks in relation to international seafood trade (33).

All variables (gray and blue text in *SI Appendix*, Fig. S6) were normalized on a scale between 0 and 1 at each stage of the framework to ensure equal weighting, regardless of initial units. For exposure and sensitivity, this scaling was reversed for those variables that initially had high values equaling low exposure or sensitivity (blue text in *SI Appendix*, Fig. S6). For adaptive capacity, scaling was reversed for those variables that initially had high values equaling low adaptive capacity (blue text in *SI Appendix*, Fig. S6).

#### Exposure.

Nutrient supplies from fisheries will be affected by changes to foreign fishing access agreements, for example, through loss of access to a particular EEZ, and by changes in trade patterns. Exposure to foreign fishing changes (E_FF_) was calculated from the mean of two normalized subvariables: variability in foreign fishing catches over time (CvFF) and diversity of locations where a country undertakes foreign fishing (DivFF). Variability in foreign fishing catches over time (CvFF) was estimated from the coefficient of variation in foreign fishing catches over time. The coefficient of variation was estimated from tonnage of foreign fishing catches by each nation from 1976 to 2015 (*SI Appendix*, Table S4). The inference is that a high coefficient of variation represents greater volatility in the access to foreign fishing catches and thus greater exposure to changes in nutrient supplies. The location diversity for foreign fishing (DivFF) was estimated using the Shannon Weaver index in the vegan package in R (57) and averaged across the seven nutrients used in the earlier analyses (*SI Appendix*, Table S4). Low values indicate most of the nutrients caught by a nation’s foreign fishing fleet are being sourced from few locations, whereas high values indicate the nutrient catch from a nation’s foreign fishing fleet is spread more evenly across many locations. The inference is that a high diversity of locations will help mediate volatility in foreign fishing catch and changes to foreign fishing access agreements, as loss of access to one EEZ (or high seas) is unlikely to have a large impact on nutrient supplies.

Exposure to changes in imports of nutrients (E_IMP_) was calculated from the mean of two normalized subvariables: variability in imports over time (CvIMP) and the diversity of nations suppling imports (DivIMP). Variability in imports (CvIMP) was estimated from the coefficient of variation in imports over time. The coefficient of variation was estimated from tonnage of imports by each nation from 1976 to 2015 (*SI Appendix*, Table S4). The inference is that a high coefficient of variation represents greater volatility in imports and thus greater exposure to changes in nutrient supplies. The diversity of nations supplying imports (DivFF) was estimated using the Shannon Weaver index and averaged across the seven nutrients used in the earlier analyses (*SI Appendix*, Table S4). Low values indicate most of the nutrients imported by a nation are being sourced from few countries, whereas high values indicate the nutrients imported by a nation are sourced more evenly from many countries. The inference is that high diversity of import sources will help mediate volatility in imports as loss of supplies from one source is unlikely to have a large impact on overall nutrient supplies.

To account for variability in the relative importance of foreign fishing and trade to different nations, exposure to changes in foreign fishing (E_FF_) and imports of nutrients (E_IMP_) were weighted by the contribution of foreign fishing (F_FF_) and imports (F_IMP_), respectively, to a nation’s fishery-derived nutrient supply (including foreign fishing, trade, and domestic supply) in relation to the seven nutrients focused on in this study (*SI Appendix*, Table S4). As a result, the exposure metrics were down weighted if foreign fishing and imports contributed little to national nutrient supply, e.g., if domestic catch dominated supply. These weightings also used national catches adjusted to remove catches from FOC vessels. Total exposure for each country was then estimated from the mean of the constituent exposure metrics (weighted E_IMP_ and E_FF_; *SI Appendix*, Table S4). The results were mapped using the rnaturalearth package in R.

#### Sensitivity.

The sensitivity of a nation to changes in supplies of fishery-derived nutrients from foreign fishing and trade will depend on how dependent the population is on fish for food and the prevalence of micronutrient deficiencies. Thus, sensitivity was calculated from the mean of two normalized subvariables: the proportion of animal protein supplied by fish (S_FD_; *SI Appendix*, Table S5) and the prevalence of inadequate micronutrient intake index (S_PIMII_; mean of 14 micronutrients [calcium, copper, iron, folate, magnesium, niacin, phosphorus, riboflavin, thiamin, vitamin A, vitamin B12, vitamin B6, vitamin C, and zinc]) (18) (*SI Appendix*, Table S5). While this list of micronutrients differs slightly from those used in the exposure metric, it provides a broader picture of prevalence of inadequate levels of micronutrient intake and thus the sensitivity of the country to changes in nutrient supplies. Overall availability of adequate energy may also impact on national sensitivity to changes in fishery-derived nutrients (58). However, access to adequate energy was strongly correlated with S_PIMII_ (Spearman Rank correlation of −0.7; *SI Appendix*, Fig. S10), so we did not include this index in the sensitivity metric. The results were mapped.

#### Adaptive capacity.

Finally, the capacity of a nation to adapt to changing nutrient supplies from fisheries will depend on many latent factors that we have estimated using a series of measurable proxies, including socioeconomic status, health care, and food trade balance. Thus, adaptive capacity was estimated from the mean of three normalized metrics: socioeconomic status (AC_SE_), expenditure on health care (AC_N_), and status as a food importer (AC_IMP_). The results were mapped.

The socioeconomic metric (AC_SE_) was calculated from the mean of two normalized subvariables (*SI Appendix*, Table S4): GDP per capita (AC_GDP_) and conflict and political stability (AC_S_) (*SI Appendix*, Table S5). GDP per capita (59) was used to indicate the economic capacity of the country to adapt to changing nutrient supplies from marine fisheries. Conflict and political instability are drivers of food insecurity (60); thus, an index of political stability and the absence of violence was also included (58). Inequality can severely impact food security (2, 60). However, the coefficient of human inequality, a measure of inequalities in education, health care, and income (59), was strongly correlated with GDP per capita (Spearman rank correlation of 0.8; *SI Appendix*, Fig. S11) so we did not include this index in the socioeconomic metric.

Health care expenditure (AC_HE_) as a percentage of GDP (59) (*SI Appendix*, Table S4), was used as a proxy of health care and thus the ability of the nation to either address inadequate nutrition or the health consequences of inadequate nutrition (*SI Appendix*, Table S5). Status of each nation as a net importer of food (AC_IMP_; *SI Appendix*, Table S4) was used as the third component of adaptive capacity as it indicated the broader capacity of the nation to counter changes in imports of fishery-derived nutrients with domestic production. The inference is that being a net importer would reduce adaptive capacity.

### Vulnerability of Nutrient Supply to Changes in Foreign Fishing, Trade, and Climate.

Food and nutrient supply are not only affected by foreign fishing and trade arrangements, they are also affected by climate variability (60). Indeed, trade and climate change have been identified as two of the core drivers undermining global efforts to address malnutrition (60). Therefore, we recalculated national vulnerability to changes in nutrient supplies by adding a metric of exposure to climate change–driven shifts in nutrient supplies. Exposure to climate change (*E_CC_*) was calculated from the mean of two normalized subvariables: predicted change in domestic catch from 2010 to 2050 (*CpDC*) and species diversity of domestic catch weighted by nutrient supply (*DivDC*) (*SI Appendix*, Table S4).

Past research assessing the exposure of fisheries to climate change has used changes in temperature to 2050, e.g., ref. 6. However, this indicator does not directly reflect changes in the productivity of fisheries; thus, in this study, the exposure of countries to changes in climate (*E_CC_*) was estimated from projected mean relative change in maximum potential fish production from 2010 to 2050 (*CpDC*), under the business-as-usual representative concentration pathway (RCP) scenario (RCP 8.5). These estimates were obtained from ref. 40 (*SI Appendix*, Table S5), which used the marine model intercomparison project model ensemble outputs, consisting of four marine ecosystem models forced by two Earth system models. Model outputs were mapped and spatially averaged to each country’s EEZ. The inference is that large declines in domestic catch production represent greater exposure to declines in nutrient supplies due to climate change.

The species diversity of domestic catch weighted by nutrient supply (*DivDC*) was estimated using the Shannon Weaver index and averaged across nutrients (*SI Appendix*, Table S4). Low values indicate most of the nutrients caught by a nation’s domestic fleet are being sourced from a few species, whereas high values indicate the nutrient catch from a nation’s domestic fleet are spread more evenly across many species. The inference is that a high diversity will help mediate declines in domestic catch as declines in one species are unlikely to have a large impact on nutrient supplies from domestic catches.

To account for variability in the relative importance of domestic catch to different nations, exposure to climate change (*E_CC_*) was weighted by the contribution of domestic catch to fisheries-derived nutrient supply (F_DC_; *SI Appendix*, Table S4).

## Supplementary Material

Supplementary File

Supplementary File

## Data Availability

The international trade data are from ref. 10. These data were not previously available publicly but are now available for download at https://doi.org/10.6084/m9.figshare.13448717.v1. Catch data are available from https://doi.org/10.4226/77/5a65572655f73 (47). Fish trait data are available from http://www.fishbase.org (48). Data and code for the Bayesian hierachical model used to produce the nutrient estimates are available from https://github.com/mamacneil/GlobalFishNutrients (7). Data on prevalence of FOCs are available from ref. 19. Data of predicted change in fisheries production due to climate change are available from https://doi.org/10.6084/m9.figshare.13475739 (40). Data on levels of inadequate nutrient intake are available from ref. 18. Indicators of socioeconomic, health, and food security are available from ref. 59 (https://hdr.undp.org/en/data), ref. 58 (https://www.fao.org/faostat/), and ref. 61 (https://unctadstat.unctad.org/EN/). Data on RNI are available from refs. 62–64.
