# Supplementary material for: Trade and foreign fishing mediate global marine nutrient supply
Source: Proc Natl Acad Sci U S A. 2022 May 23;119(22):e2120817119. doi: 10.1073/pnas.2120817119 (PMC9295801; doi:10.1073/pnas.2120817119)
Supplement: Supplementary File [file pnas.2120817119.sapp.pdf]

PNAS

Supplementary Information for

Trade and foreign fishing mediate global marine nutrient supply

Kirsty L. Nash, M. Aaron MacNeil, Julia L. Blanchard, Philippa J. Cohen, Anna K. Farmery, Nicholas A.J. Graham, Andrew L. Thorne-Lyman, Reg A. Watson, Christina C. Hicks

Corresponding Author: Kirsty Nash
Email: nashkirsty@gmail.com

This PDF file includes:

Supplementary text – Glossary
Supplementary text – Materials and Methods
Figures S1 to S12
Tables S1 to S5
Legends for Datasets S1 to S2
SI References

Other supplementary materials for this manuscript include the following:

Datasets S1 to S2

Supplementary Information Text

Glossary

Access right: Right to access waters for fishing purposes – may be through national ownership or foreign fishing access agreements.

Beneficial owner: Who owns and benefits from a vessel and its operations. Term used in relation to Flags of Convenience where the 'beneficial owner' lives in a different country to the flagged nationality of the vessel.

Bioavailable: Nutrient is in a form that is easily absorbed by the body.

Catch reconstruction: Process of reconstructing fisheries catches from a range of data sources (such as reported catches, grey literature, species ranges, local experts) and interpolation of missing data.

Circular trade flow: Where a commodity is imported from a country and the exported again to the source country.

Commodity: A resource than can be bought and sold. In the case of seafood, commodities may take the form of whole fish or derived products e.g. canned goods, fish meal etc.

Commodity groups: A group of similar commodities.

Family-level: Used where fish or derived products are identified in a dataset to family e.g. identified as belonging to the snapper family Lutjanidae. Indicates that information on the genus or species of the fish or derived product is not provided.

Flag of convenience (FOC): A practice whereby the owner (see beneficial owner) lives in a nation other than that in which their vessel is registered. E.g. the vessel's owner lives in China but the vessel is flagged as Ghanian. May be associated with illegal and unreported fishing.

Foreign fishing: Fishing carried out on the high seas or in the Exclusive Economic Zone of another nation.

Genus-level: Used where fish or derived products are identified in a dataset to genus e.g. identified as belonging to the true tuna genus *Thunnus*. Indicates that information on the species of the fish or derived product is not provided.

High seas: Parts of the ocean that are not included in the territorial waters of a nation.

Micronutrient: Vitamins and minerals that are essential to our diet in very small quantities.

Nutrient yield: The annual mass of nutrients divided by the population size and days in the year to give mass per capita per day.

Recommended nutrient intake (RNI): The nutrient intake necessary to meet the basic nutritional needs of over 97.5% of the population. Note that different RNIs may be set for different demographics e.g. an RNI for reproductive aged women (19-50 years) as used in this study.

Reexport: Where a commodity is imported and then exported again. This may occur after processing into another form.

Species-level: Used where fish or derived products are identified in a dataset to species e.g. identified as an Atlantic Salmon *Salmo salar*.

Taxonomic grouping: Fish or commodities are grouped according to taxonomy e.g. identified to species or to a lower resolution, e.g to family. In some cases very broad groupings such as marine fin fish may be used when finer scale taxonomic information is missing.

Taxonomic identity: Fish or commodities classified according to taxonomy, e.g. identified as an Atlantic Salmon *Salmo salar* or identified as belonging to the true tuna genus *Thunnus*.

Taxonomic resolution: The resolution of any taxonomic identity assigned to a fish or commodity, i.e. species, genus, family or a lower resolution taxonomic group.

Transshipment: Fish caught by one vessel is transferred to another vessel for transport to port.

Supplementary Methods

Catch data. We used the catch database of Watson and Tidd (1). This data source is strongly correlated (Spearman rho = 0.95) to landings data from the Sea Around Us project (2), another commonly used global catch database based on country-by-country reconstructions (Fig. S11) (3). Our database of choice was also closely linked to official reporting by the UN's FAO and its associated regional organizations. However, these reported landings can underestimate total landings for a variety of reasons, which include reporting failures and illegal activity.
Spatial mapping of the catch uses a process of matching the distribution of reported taxa, the access right and fishing patterns of fleets and the reported areas from a wide range of publicly available sources. This mapping was improved by using fine scale regional reporting such as that provided by tuna Regional Fisheries Management Organizations (RFMOs) and observed patterns from satellite data of Global Fishing Watch's (GFW) vessel Automatic Identification System (AIS)-based data (4).

Trade data. We used the trade data of Watson, Nichols, Lam and Sumaila (5). This database uniquely links fisheries capture data (described above) to seafood exports and imports reported by FAO (6). Flows of fish in the global seafood trade are then traced in tonnes by matching commodity groups to taxa and trading partners for seafood from UN's annual Comtrade data (1988–2015) (7). Where no information on trade was available, WTO's primary trading partner data were used (8).

Interpolation of nutrient data. Where catch and trade information were provided at taxonomic resolutions below family, and therefore nutrient concentrations were not available from the hierarchical model, the mass of nutrients was estimated using a four-tier interpolation approach. This process is described below using the example of Spain sourcing from Namibia:

1. We used the median nutrient concentration weighted by tonnage for all taxa of known nutrient value caught in Namibia by Spain (for catch data interpolation) or exported from Namibia to Spain (for trade data interpolation).
2. If the above data were not available, we used the median nutrient concentration weighted by tonnage for all taxa of known nutrient value caught in Namibia by all countries (for catch data interpolation) or exported from Namibia to all countries (for trade data interpolation).
3. Where these data were not available, for trade data interpolation, we used the estimated median nutrient concentration weighted by taxa caught in Namibia by all countries.
4. Finally, if the above data were not available, we used the estimated median nutrient concentration weighted by tonnage for all taxa of known nutrient value caught in all countries (interpolation of catch data) or exported from all countries (interpolation of trade data).

The percentage of tonnage interpolated varied widely among nations (Dataset S2); the mean percentage across all nations was 24% for domestic catch data, 10% for foreign fishing data, and 25% for trade data.

Sensitivity Analyses. The resolution of the catch and trade data varied widely among nations (Dataset S2). The hierarchical model was only used to estimate nutrient concentrations for those taxa identified to family, genus or species level. The nutrient content of lower resolution taxa was estimated using interpolation. To understand the potential impact of this interpolation on the vulnerability calculations we recalculated national vulnerability to changes in fishery-derived nutrient supplies in two ways: 1) only including those countries where at least 75% of the caught and traded tonnage was identified to family, genus or species level; and 2) only including data

identified to family, genus or species level. We then explored the relationships between these different vulnerability estimates.

When the calculations only included countries with >75% of caught and traded tonnage identified to family, genus or species level, the estimated vulnerabilities were all slightly higher for these nations compared with their vulnerabilities when all nations were included (Fig. S12A-B). When only data identified to family, genus or species level were used, the estimated vulnerabilities showed more variation but followed a similar, bimodal distribution to the vulnerability estimates including all the data (Fig. S12C-D).

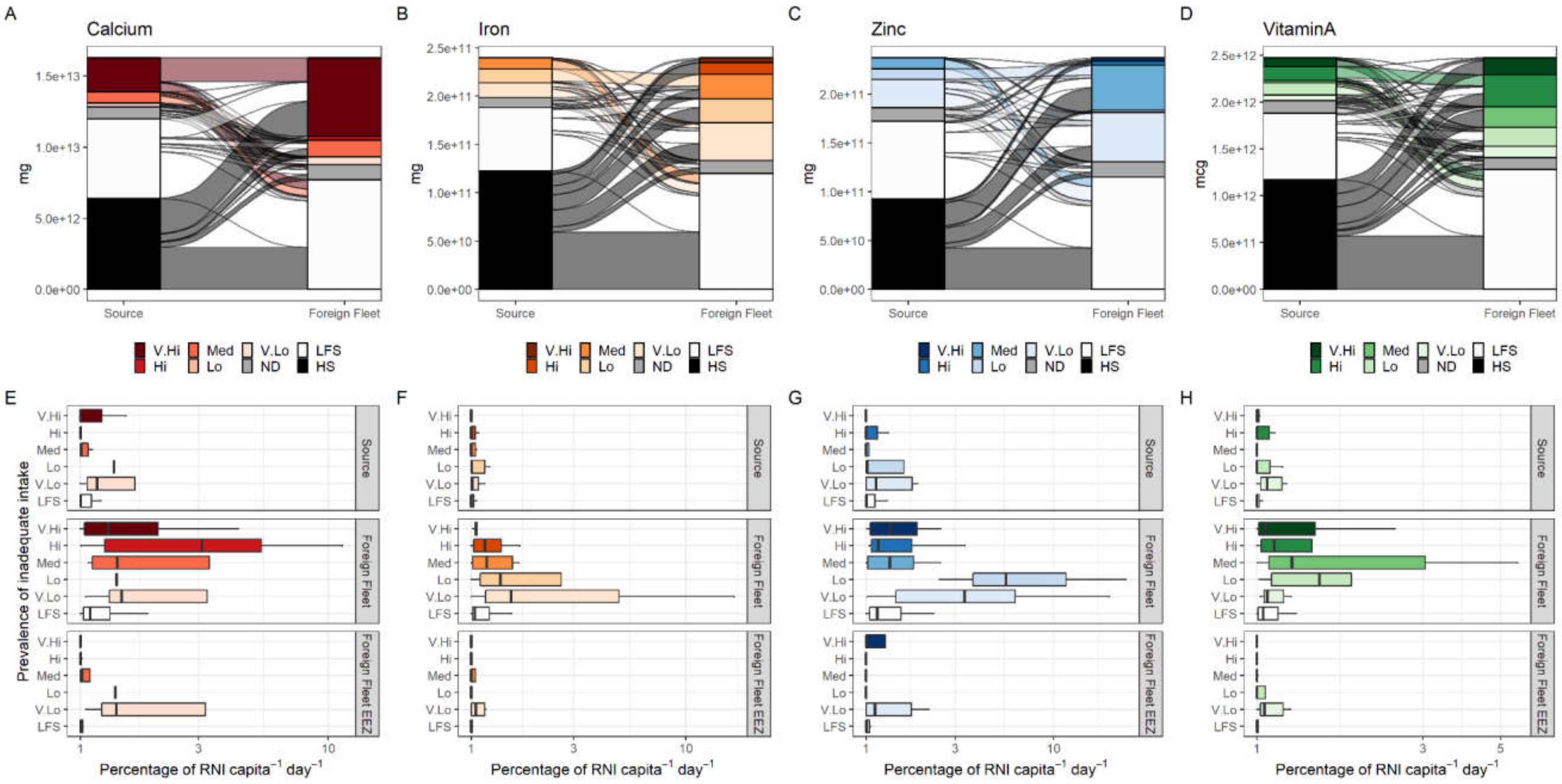

Fig. S1. Annual flow and yield of fishery-derived nutrients due to foreign fishing, highlighting the nutrient status of countries dependent on fish for food (>10% of animal source protein from fish). (A-D) Flow of calcium (mg), iron (mg), zinc (mg), and vitamin A (mcg) due to foreign fishing for nations grouped by prevalence of inadequate intake of respective nutrients in source countries and foreign fleet (sink) countries. (E-H) Top panels show yields (log10 percentage of RNI capita^{-1} day^{-1}) extracted from source nation's EEZs grouped by source nations' nutrient intake. Middle panels show yields caught by foreign fleets in EEZs and the high seas, grouped by foreign fleet (sink) nations' nutrient intake. Bottom panels only include catches from foreign EEZs. Prevalence of inadequate nutrient intake categories: V.Hi (dark shading) – countries with >50% of population with inadequate intake of respective nutrient.; Hi – 25<%≤50; Med – 10<%≤25; Lo – 5<%≤10; V.Lo (light shading) – ≤5%; ND (grey shading) – no deficiency data available for these nations; LFS (white shading) - <10% of animal sourced protein from fish; HS (black shading) – nutrients sourced from high seas.

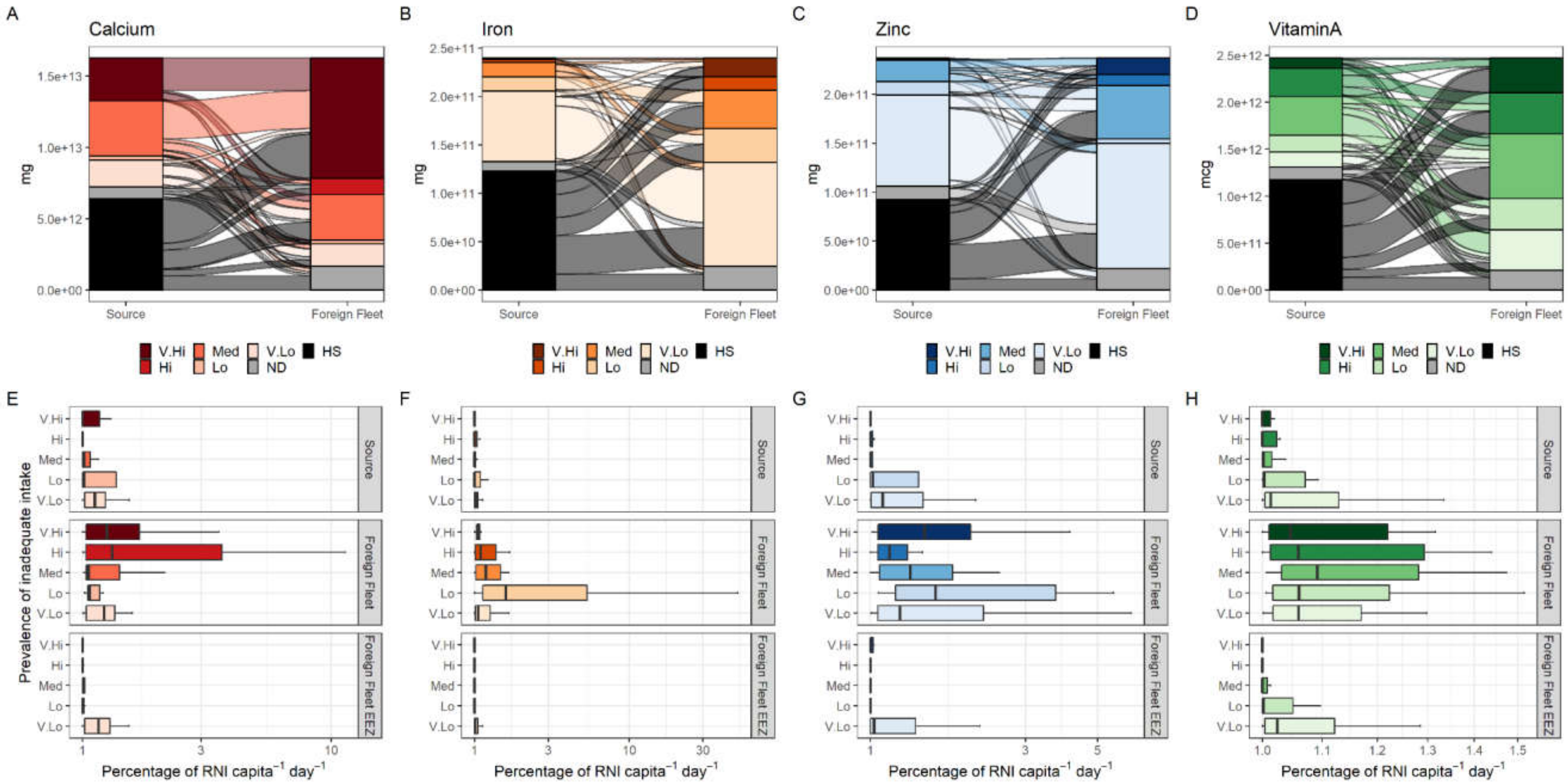

Fig. S2. Annual flow and yield of fishery-derived nutrients due to foreign fishing for all nations and not accounting for flags of convenience. (A-D) Flow of calcium (mg), iron (mg), zinc (mg), and vitamin A (mcg) due to foreign fishing for nations grouped by prevalence of inadequate intake of respective nutrients in source countries and foreign fleet (sink) countries. (E-H) Top panels show yields (log10 percentage of RNI capita^{-1} day^{-1}) extracted from source nation's EEZs grouped by source nations' nutrient intake. Middle panels show yields caught by foreign fleets in EEZs and the high seas, grouped by foreign fleet (sink) nations' nutrient intake. Bottom panels only include catches from foreign EEZs. Prevalence of inadequate nutrient intake categories: V.Hi (dark shading) – countries with >50% of population with inadequate intake of respective nutrient.; Hi – 25<%≤50; Med – 10<%≤25; Lo – 5<%≤10; V.Lo (light shading) – ≤5%; ND (grey shading) – no deficiency data available for these nations; HS (black shading) – nutrients sourced from high seas.

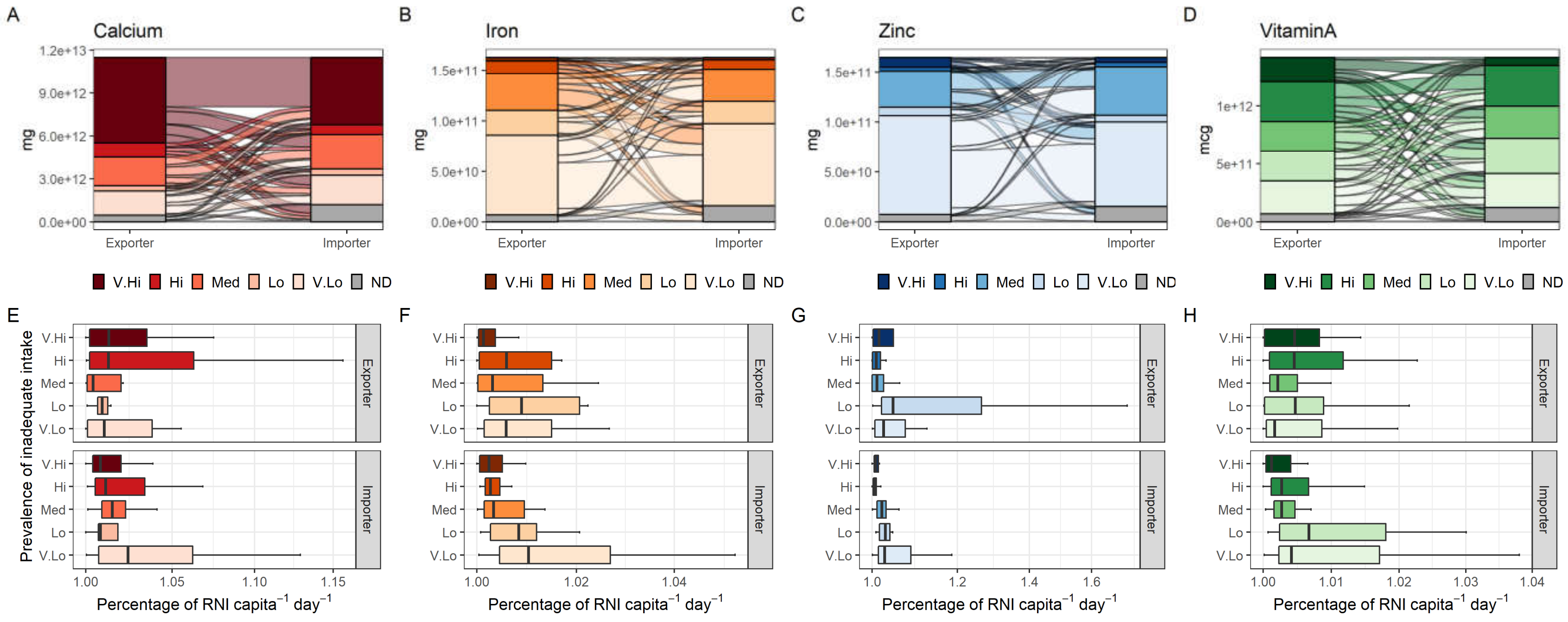

Fig. S3. Annual flow and yield of fishery-derived nutrients due to international trade. (A-D) Flow of calcium (mg), iron (mg), zinc (mg), and vitamin A (mcg) due to trade for nations grouped by prevalence of inadequate intake of respective nutrients in source (exporter) and sink (importer) countries. (E-H) Top panels shows yields (log10 percentage of RNI capita^{-1} day^{-1}) exported from source nation, grouped by source nations' nutrient intake. Bottom panels show yields imported by sink countries, grouped by sink nations' nutrient intake. Prevalence of inadequate nutrient intake categories: V.Hi (darkest shading) – countries with >50% of population with inadequate intake of respective nutrient.; Hi – 25<%≤50; Med – 10<%≤25; Lo – 5<%≤10; V.Lo (lightest shading) – ≤5%.

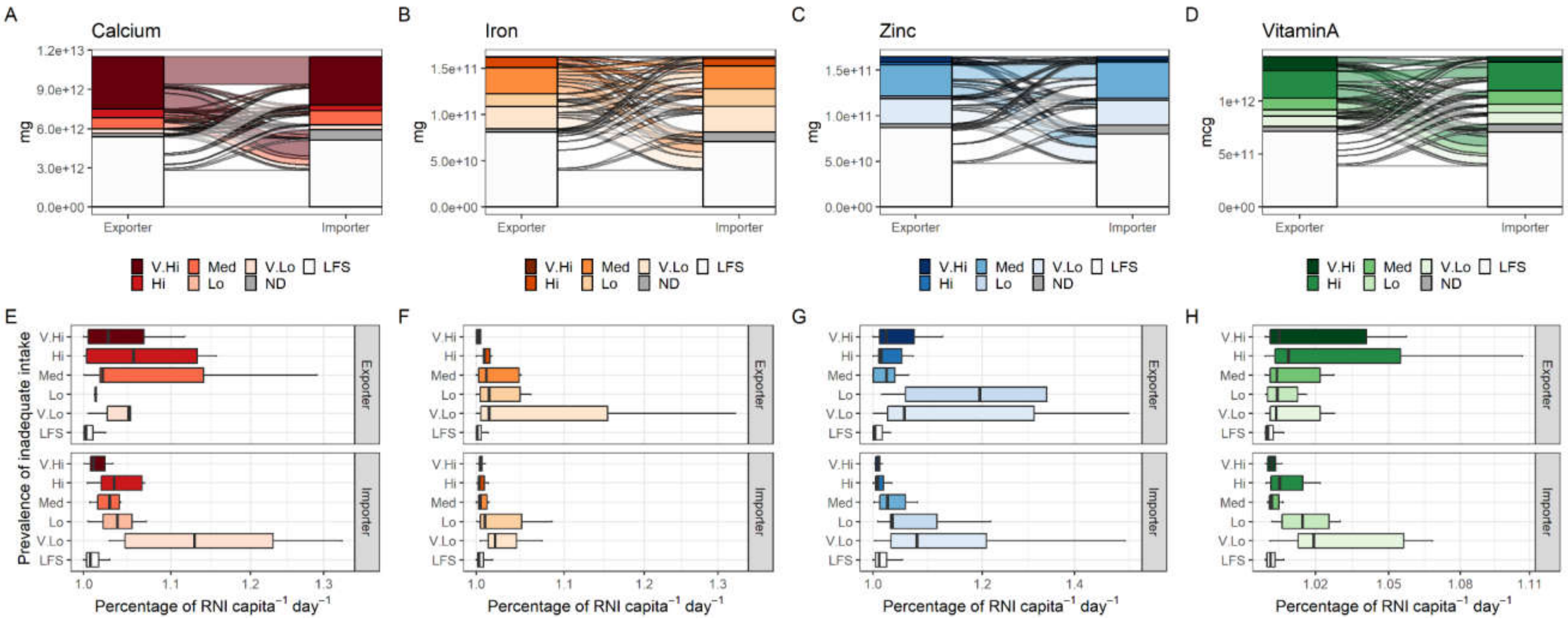

Fig. S4. Annual flow and yield of fishery-derived nutrients due to international trade, highlighting the nutrient status of countries dependent on fish for food (>10% of animal sourced protein from fish). (A-D) Flow of calcium (mg), iron (mg), zinc (mg), and vitamin A (mcg) due to trade for nations grouped by prevalence of inadequate intake of respective nutrients in source (exporter) and sink (importer) countries. (E-H) Top panels shows yields (log10 percentage of RNI capita^{-1} day^{-1}) exported from source nation, grouped by source nations' nutrient intake. Bottom panels show yields imported by sink countries, grouped by sink nations' nutrient intake. Prevalence of inadequate nutrient intake categories: V.Hi (darkest shading) – countries with >50% of population with inadequate intake of respective nutrient.; Hi – 25<%≤50; Med – 10<%≤25; Lo – 5<%≤10; V.Lo (lightest shading) – ≤5%; LFS (white shading) - <10% of animal sourced protein from fish.

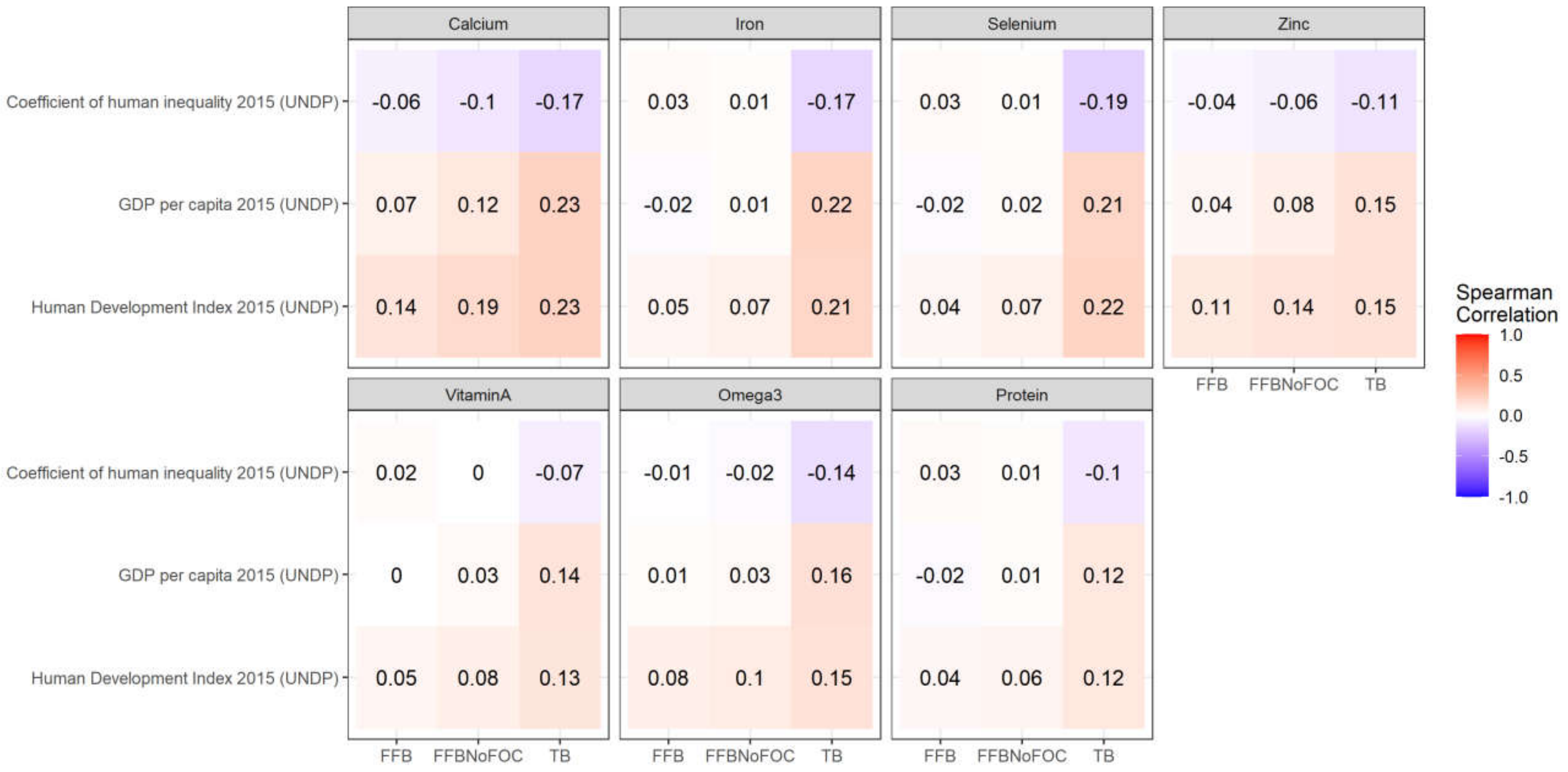

Fig. S5. Spearman rank correlations of trade and foreign fishing balance against metrics of socio-economic status. Shading represents strength of correlations. FFB is foreign fishing balance - nutrient gains by a nation's own fleet fishing in foreign waters or high seas minus losses to other nations' foreign fleets fishing in own EEZ (measured in number of RNI). FFBNoFOC is foreign fishing balance when flags of convenience are accounted for. TB is trade balance – nutrient gains from imports minus nutrient losses through exports (measured in number of RNI). Year and source of socio-economic data are provided.

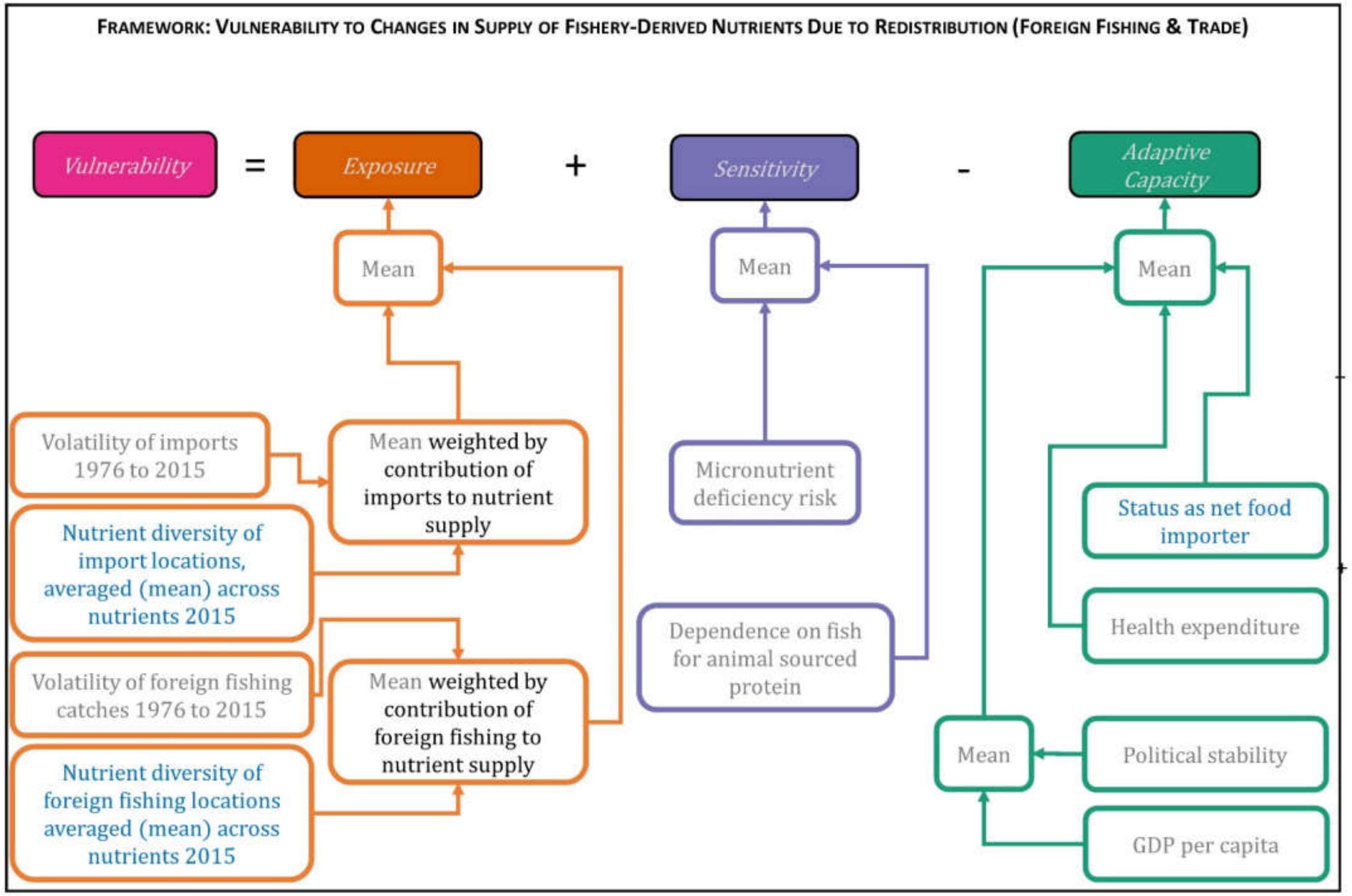

Fig. S6. Conceptual framework used to estimate vulnerability to changes in supply of fishery-derived nutrients due to foreign fishing and trade. Framework details constituent exposure, sensitivity and adaptive capacity indices and scaling. Metrics in blue and grey text were scaled from 0 to 1. For those metrics shown in blue text, values were reversed for rescaling to ensure high values equalled high exposure, sensitivity or adaptive capacity. Weightings of imports or foreign fishing used in exposure metric account for imports/foreign fishing relative to total nutrient supply from imports, foreign fishing and domestic catch. See Table S4 for more information.

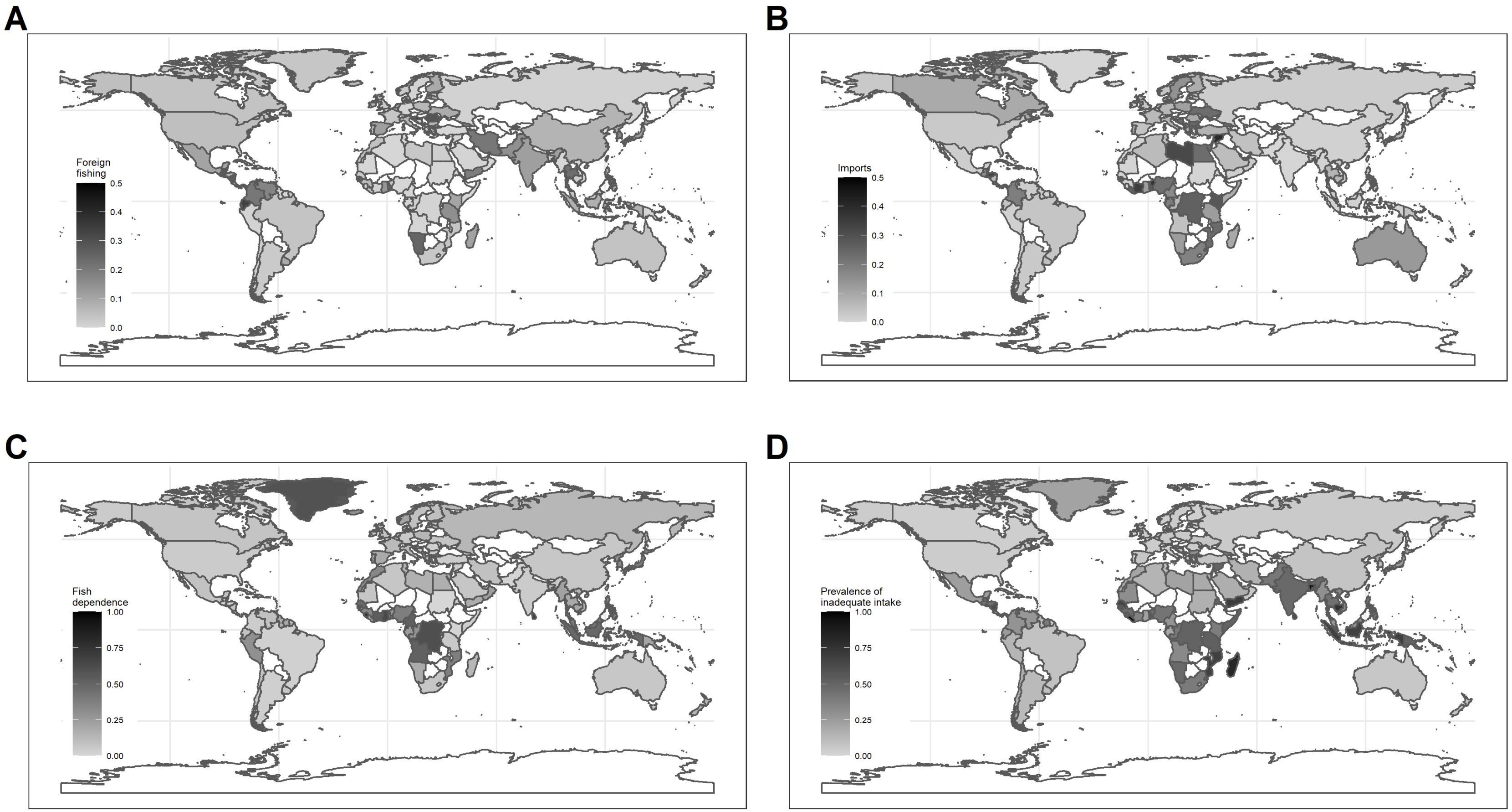

Fig. S7. National exposure and sensitivity to changes in fisheries-derived nutrient supply due to changes in foreign fishing and trade. Exposure to changes in (A) foreign fishing and (B) imports. Sensitivity due to (C) dependence on fish for animal-sourced protein and (D) prevalence of inadequate nutrient intake. Scales in A and B only go up to 0.5 as these metrics were weighted by relative contribution of foreign fishing or imports to national nutrient flows. Catches flowing to vessels using flags of convenience were not included in the exposure estimates.

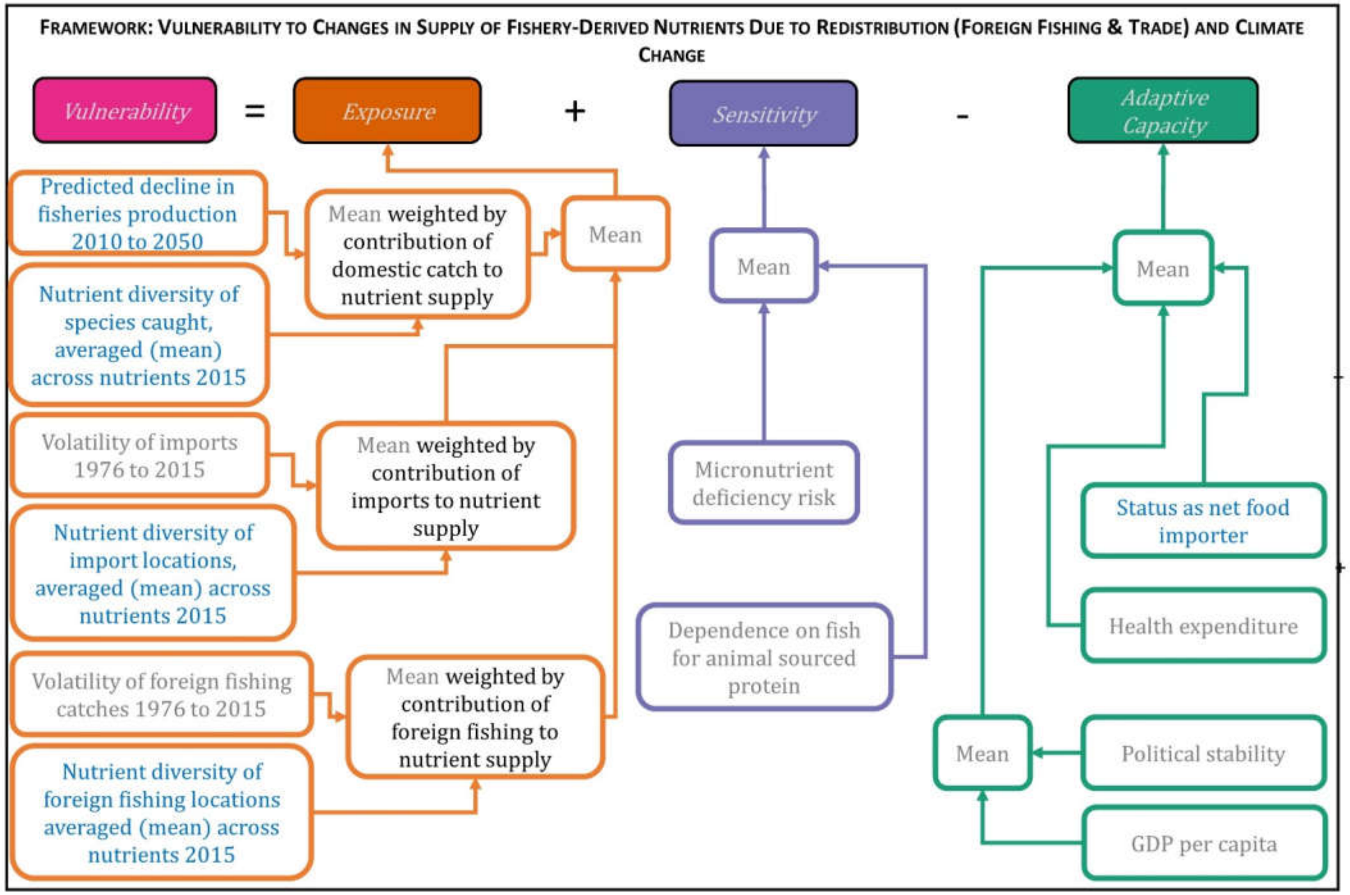

Fig. S8. Conceptual framework used to estimate vulnerability to changes in supply of fishery-derived nutrients due to foreign fishing, trade and climate change. Framework details constituent exposure, sensitivity and adaptive capacity indices and scaling. Metrics in blue and grey text were scaled from 0 to 1. For those metrics shown in blue text, values were reversed for rescaling to ensure high values equalled high exposure, sensitivity or adaptive capacity. Weightings of imports or foreign fishing used in exposure metric account for imports/foreign fishing relative to total nutrient supply from imports, foreign fishing and domestic catch. See Table S4 for more information.

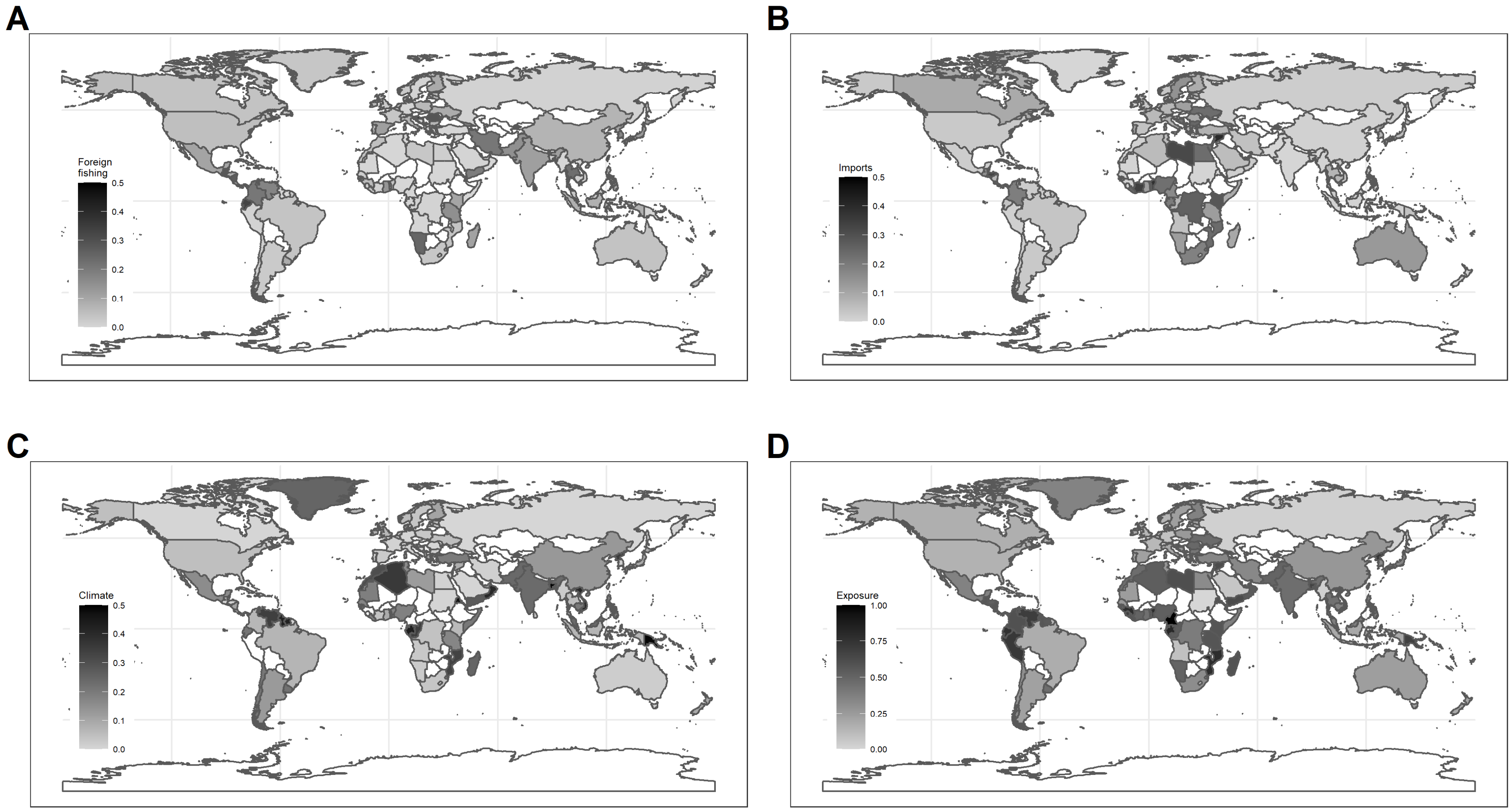

Fig. S9. National exposure to changes in fisheries-derived nutrient supply due to changes in foreign fishing, trade and climate. Exposure to changes in (A) foreign fishing, (B) imports, (C) climate induced changes in domestic fisheries productivity. (D) combined exposure metric. Scales in A-C only go up to 0.5 as these metrics were weighted by relative contribution of foreign fishing, imports or domestic catch to national nutrient supplies.

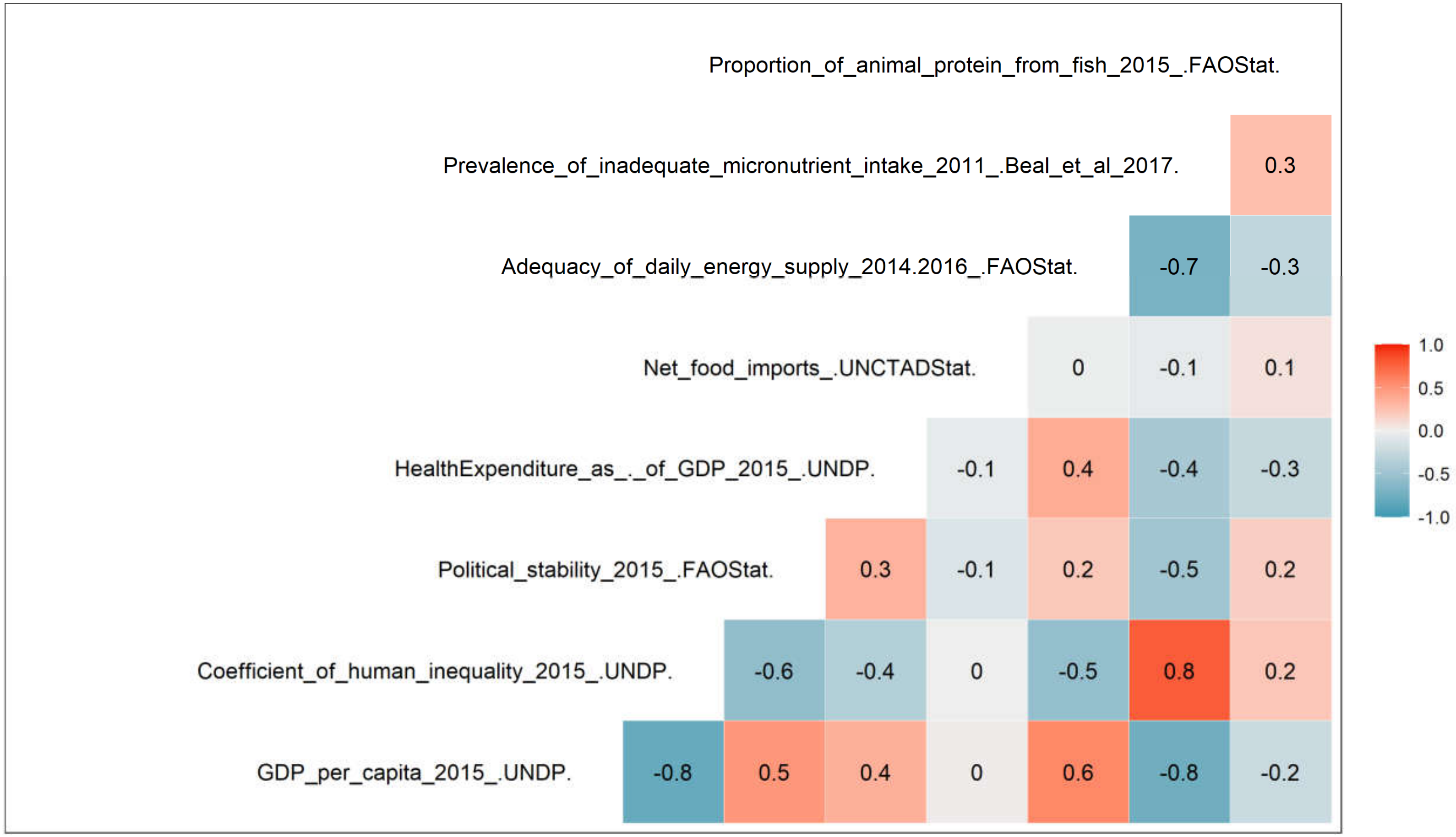

Fig. S10. Spearman rank correlations of sensitivity and adaptive capacity indicators. Shading represents strength of correlations. Year and source of data are provided.

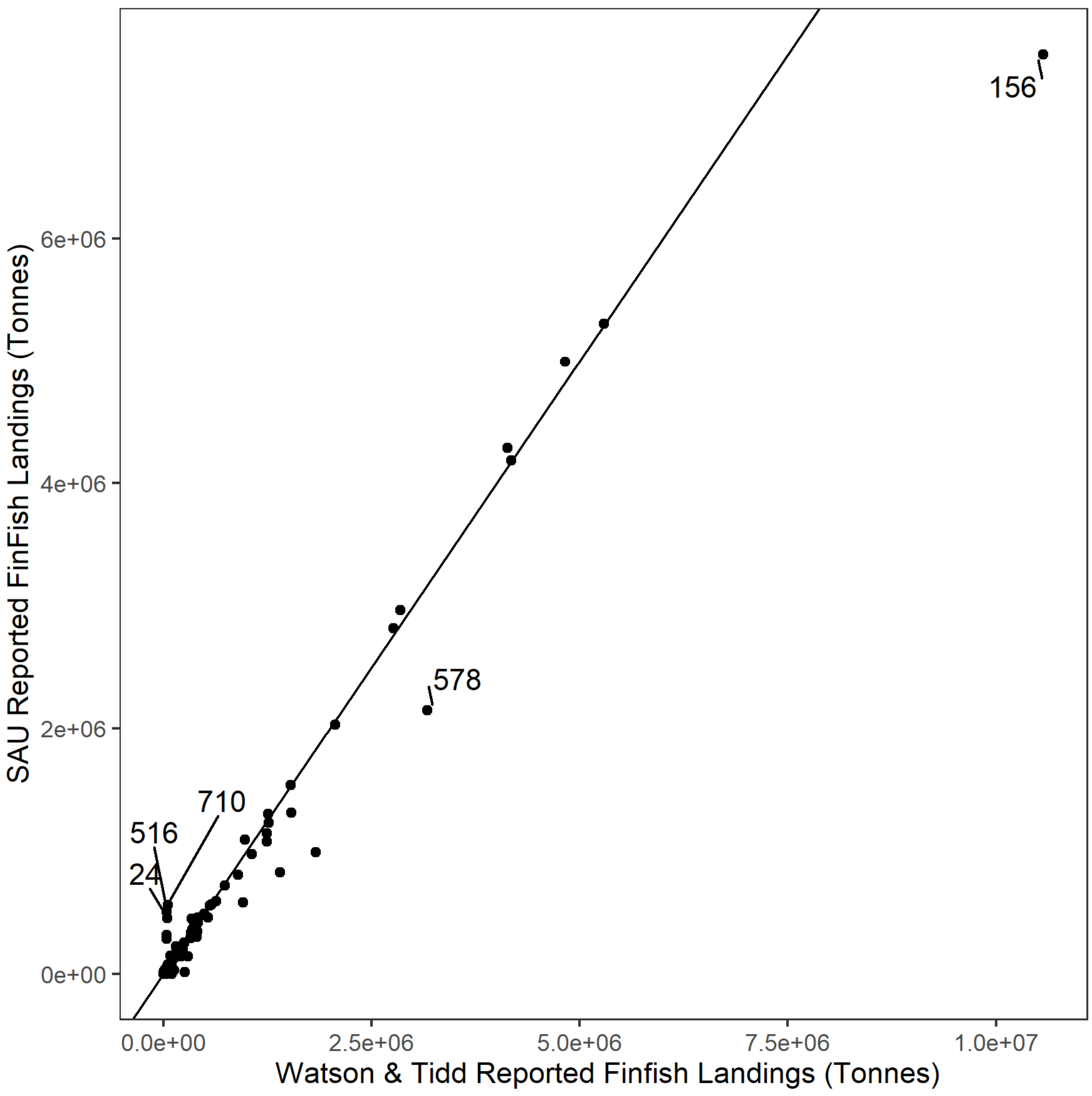

Fig. S11. Comparison of reported tonnage of finfish landings by fishing country in 2015 from Watson and Tidd (1) and Sea Around Us (2) databases. Spearman rank correlation of 0.95. Countries labelled: 24 – Angola; 156 – China; 516 – Namibia; 578 – Norway; 710 – South Africa.

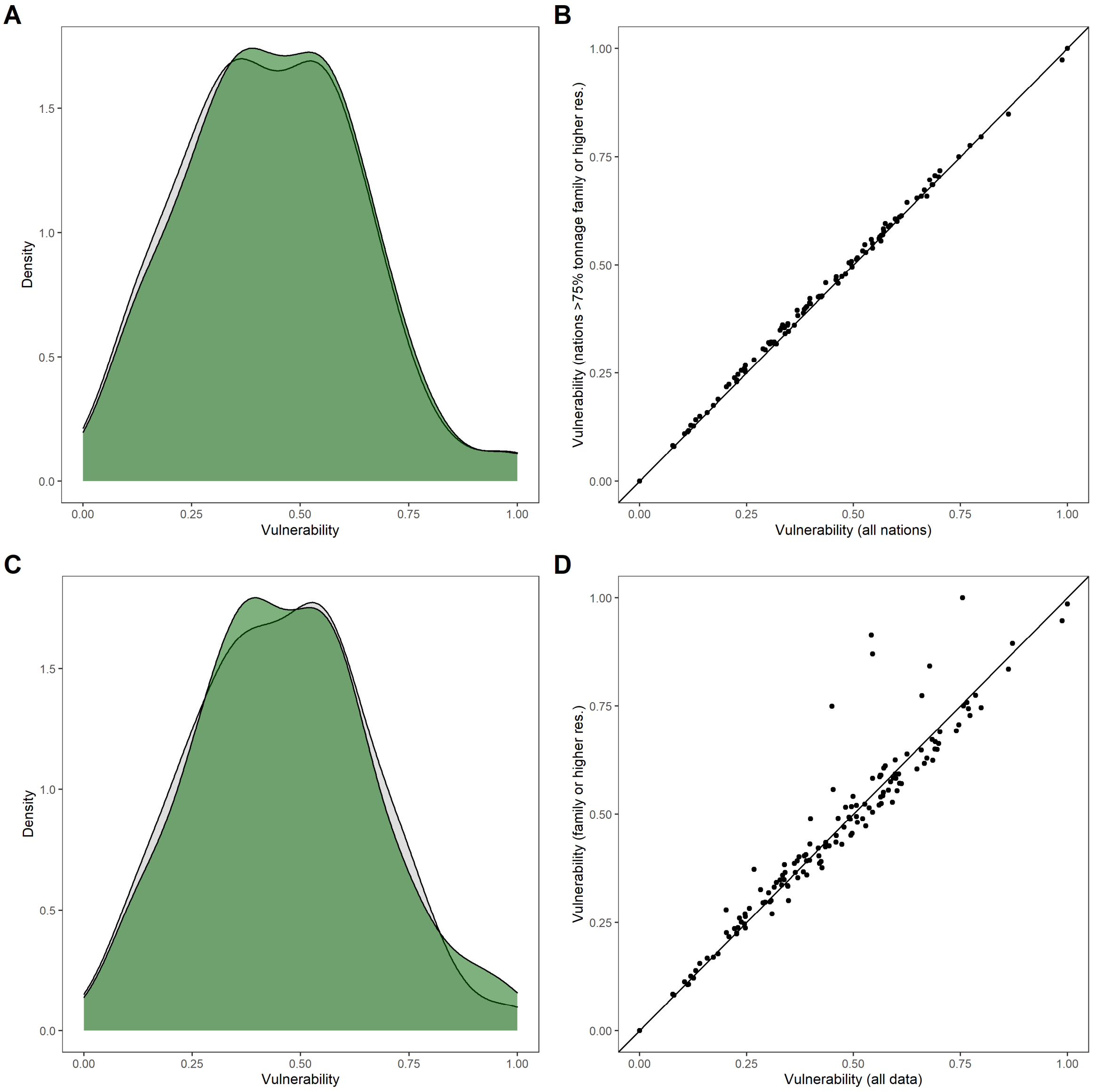

Fig. S12. Sensitivity analyses exploring impact of taxonomic resolution on vulnerability assessment. (A-B) Vulnerability when all nations included in assessment (grey shading in a, x-axis in b) compared to vulnerability when only those nations with >75% of catch or trade identified to species, genus or family level are included (green shading in A, y-axis in B). Only those nations present in both datasets are plotted. (C-D) Vulnerability when all data included in assessment (grey shading in c, x-axis in d) compared to vulnerability when only catch and trade data identified to species, genus or family level are included (green shading in C, x-axis in D).

Table S1. Recommended nutrient intake (RNI) values for reproductive age females (19-50 years) who are not pregnant or lactating. RNI values used to estimate the number of RNIs that could be met by nutrient supplies from trade and foreign fishing and the percentage of RNI per capita flowing through trade and foreign fishing. Note there is no RNI for Omega-3. In this instance, the Adequate Intake (AI) value was used. Reproductive age females were used because this demographic experience high rates and impacts of undernutrition (9, 10).

**Nutrient**	**Demographic**	**RNI**	**Source**
Calcium	Females 19-50 yrs	1000mg day^{-1}	(11)
Iron	Females 19-50 yrs	29.4mg day^{-1} (assuming 10% bioavailability)	(11)
Selenium	Females 19-50 yrs	26µg day^{-1}	(11)
Zinc	Females 19-50 yrs	4.9mg day^{-1} (assuming moderate bioavailability)	(11)
Vitamin A	Females 19-50 yrs	500 µg RE day^{-1}	(11)
Omega-3	Females 19-50 yrs	1.1g day^{-1}	(12)
Protein	Females 19-50 yrs	41g day^{-1} (assuming body weight of 62kg)	(13)

Table S2. Proportion of countries showing positive, negative or neutral trade and foreign fishing balances. (A) Balances in relation to either foreign fishing or trade. (B) Balances in relation to both foreign fishing and trade. FF – foreign fishing, excl. FOC – excluding flag of convenience vessels, excl. HS – excluding high seas.

A

Balance	Data	FF	FF excl. FOC	FF excl. HS	FF excl. FOC, & HS	Trade
-	All Data	0.31	0.31	0.6	0.61	0.36
+	All Data	0.64	0.64	0.26	0.24	0.64
0	All Data	0.05	0.05	0.14	0.14	0.01
-	SIDS	0.44	0.47	0.72	0.75	0.38
+	SIDS	0.56	0.53	0.16	0.12	0.59
0	SIDS	0	0	0.12	0.12	0.03
-	Africa	0.29	0.29	0.79	0.82	0.24
+	Africa	0.66	0.66	0.16	0.13	0.76
0	Africa	0.05	0.05	0.05	0.05	0

B

		FF excl. FOC, and Trade		FF excl. FOC, plus Trade	
**Balance**	**Data**	**Proportion**	**Quadrant in Figure 1**	**Proportion**	**Background shading Figure 1**
-	All Data	0.11	Lower left	0.28	Grey shading
+	All Data	0.40	Upper right	0.72	White shading
+/-	All Data	0.49	Upper left/lower right	NA	
-	SIDS	0.22	Lower left	0.34	Grey shading
+	SIDS	0.38	Upper right	0.66	White shading
+/-	SIDS	0.41	Upper left/lower right	0	
-	Africa	0.05	Lower left	0.26	Grey shading
+	Africa	0.47	Upper right	0.74	White shading
+/-	Africa	0.47	Upper left/lower right	NA	

Table S3. Categories of prevalence of inadequate nutrient intake and respective sample sizes. (A) Categories used for percentage of population with inadequate nutrient intake in relation to calcium (Ca), iron (Fe), zinc (Zn) and vitamin A (Vit A) for foreign fishing (Fig. 2; fig. S1-S2) and trade (fig. S3-S4) flow and yield plots. (B) Sample sizes of respective categories of prevalence of inadequate nutrient intake used in flow and yield plots.

A

Category	% Inadequate Intake
ND (No data)	No data
V.Hi (Very high)	>50%
Hi (High)	25<%≤50
Med (Medium)	10<%≤25
Lo (Low)	5<%≤10
V.Lo (Very Low)	≤5%

B

Category	Type	Group	Ca	Fe	Zn	Vit A
V.Hi	Foreign Fishing	Source	42	13	9	22
V.Hi	Foreign Fishing	Foreign Fleet	40	12	10	20
Hi	Foreign Fishing	Source	17	13	19	29
Hi	Foreign Fishing	Foreign Fleet	19	14	16	27
Med	Foreign Fishing	Source	19	18	28	22
Med	Foreign Fishing	Foreign Fleet	17	14	25	19
Lo	Foreign Fishing	Source	5	13	9	12
Lo	Foreign Fishing	Foreign Fleet	6	13	7	13
V.Lo	Foreign Fishing	Source	20	46	38	18
V.Lo	Foreign Fishing	Foreign Fleet	16	45	40	19
V.Hi	Trade	Exporter	43	11	8	21
V.Hi	Trade	Importer	50	15	11	26
Hi	Trade	Exporter	20	14	20	33
Hi	Trade	Importer	23	16	23	36
Med	Trade	Exporter	19	21	32	25
Med	Trade	Importer	21	22	33	26
Lo	Trade	Exporter	8	19	11	13
Lo	Trade	Importer	8	19	11	15
V.Lo	Trade	Exporter	23	48	42	21
V.Lo	Trade	Importer	23	53	47	22

Table S4. Indicators included in vulnerability assessments. See fig. S6&S8 for full details of the vulnerability frameworks.

Component	Indicator	Description	Method of calculation	Interpretation
**Exposure**	Variability in foreign fishing catches	Coefficient of variation estimated from total tonnage caught by a nation's foreign fishing fleet from 1976-2015	$CvFF = \frac{\sigma}{\bar{x}}$<br>σ: standard deviation of tonnage caught from 1976-2015<br>$\bar{x}$: mean tonnage caught from 1976-2015	Exposure to changes in foreign fishing supplies. A high coefficient of variation represents volatility in foreign fishing catches and thus high exposure to changes in nutrient supplies
	Diversity of foreign fishing locations	Diversity of locations estimated from nutrient tonnage using the Shannon Weaver Index	$DivFFi_n = -\sum_{l=0}^{L} p_l ln p_l$<br>*p*: proportion of foreign fishing catch of nutrient *n* by nation *i* caught in foreign location *l*<br>*L*: is the number of locations (other countries' EEZs and the high seas) in which nation fishes<br><br>Mean taken across the 7 nutrients to estimate *DivFFi*	Redundancy in nutrient supply from foreign fishing. A high diversity indicates nutrient supplies are sourced more evenly across many foreign fishing locations, so loss of access to a location likely to have a lower relative impact on overall nutrient supply
	Variability in imports	Coefficient of variation estimated from total tonnage of imports to each nation from 1976-2015	$CvIMP = \frac{\sigma}{\bar{x}}$<br>σ: standard deviation of tonnage imported from 1976-2015<br>$\bar{x}$: mean tonnage imported from 1976-2015	Exposure to changes in imports. A high coefficient of variation represents volatility in imports and thus high exposure to changes in nutrient supplies
	Diversity of import partners	Diversity of import partners estimated from nutrient tonnage using the Shannon Weaver Index	$DivIMPi_n = -\sum_{l=0}^{L} p_l ln p_l$<br>*p*: proportion of imports of nutrient *n* to nation *i*'s from country *l*<br>*L*: is the number of exporting countries supplying imports<br><br>Mean taken across the 7 nutrients to estimate *DivIMPi*	Redundancy in nutrient supply from imports. A higher diversity indicates nutrient supplies are sourced more evenly from many countries, so loss of access to imports from a country is likely to have lower relative impact on overall nutrient supply
	Predicted change in domestic catch due to climate change	Change in catch 2010 to 2050 under RCP 8.5	Sourced from (14)	Exposure to changes in domestic catch. A large negative change to production indicates a high exposure to

[only included in climate change vulnerability assessment]			decreases in nutrient supplies due to climate change
Diversity of domestic catch [only included in climate change vulnerability assessment]	Diversity of domestic catch estimated from nutrient tonnage using the Shannon Weaver Index	$$DivDCi_n = -\sum_{s=0}^{S} p_l ln p_l$$ *p*: proportion of domestic catch of nutrient *n* by nation *i* from species *s*<br>*S*: is the number of species in domestic catch<br><br>Mean taken across the 7 nutrients to estimate *DivDCi*	Redundancy in nutrient supply from domestic catch. A higher diversity indicates nutrient supplies are sourced more evenly from many species, so decline in catch of one species is likely to have lower relative impact on overall nutrient supply
Proportion of nutrient flow to nation from foreign fishing	Measure of the relative importance of foreign fishing to national supply of nutrients	$$F_{FFn} = \frac{N_{FF}}{N_{FF} + N_{LossFF} + N_{IMP} + N_{DC}}$$ N_{FF}: tonnage of nutrient *n* caught by nation through foreign fishing<br>N_{LossFF}: tonnage of nutrient *n* caught from nation's EEZ by other nations' fleets<br>N_{IMP}: tonnage of nutrient *n* imported by nation<br>N_{DC}: tonnage of nutrient *n* caught by nation's domestic fleet<br><br>Mean taken across the 7 nutrients to estimate F_{FF}	Relative importance of foreign fishing to nutrient supplies. High values indicate a high proportion of the supply of nutrients is sourced from foreign fishing
Proportion of nutrient flow to nation from trade	Measure of the relative importance of imports to national supply of nutrients	$$F_{IMPn} = \frac{N_{IMP}}{N_{FF} + N_{LossFF} + N_{IMP} + N_{DC}}$$ Mean taken across the 7 nutrients to estimate F_{IMP}	Relative importance of imports to nutrient supplies. High values indicate a high proportion of the supply of nutrients is sourced from imports
Proportion of nutrient flow to nation from domestic catch [only included in climate change	Measure of the relative importance of domestic catch to national supply of nutrients	$$F_{DCn} = \frac{N_{DC}}{N_{FF} + N_{LossFF} + N_{IMP} + N_{DC}}$$ Mean taken across the 7 nutrients to estimate F_{DC}	Relative importance of domestic catch to nutrient supplies. High values indicate a high proportion of the supply of nutrients is sourced from domestic catch

	vulnerability assessment]			
**Sensitivity**	Dependence on marine seafood	Proportion of total animal protein consumed provided by fish	$S_F = \frac{P_F}{P_A}$<br>P_F: Protein from marine finfish<br>P_A: Protein from animal sources	Sensitivity to changes in supply of fishery-derived nutrients. High values suggest high sensitivity to changes in fisheries-derived nutrient supply
	Inadequate Nutrient Intake	Prevalence of Inadequate Micronutrient Intake Index (PIMII; mean taken across 14 micronutrients)	Sourced from (15)	Sensitivity to changes in supply of fishery-derived nutrients. High values suggest high sensitivity to changes in nutrient supply
**Adaptive Capacity**	GDP per capita	GDP per capita	Sourced from (16)	Proxy for economic capacity of the country to adapt to changing nutrient supplies from marine fisheries. High values indicate high adaptive capacity to changes in nutrient supply
	Political stability	Index of political stability and the absence of violence	Sourced from (17)	Conflict and political instability are drivers of food insecurity(18, 19). High values indicate high adaptive capacity to changes in nutrient supply
	Health expenditure	Health expenditure as a percentage of GDP	Sourced from (16)	Proxy of health care and the ability of the nation to either address inadequate nutrition or the health consequences of inadequate nutrition. High values indicate high adaptive capacity to changes in nutrient supply
	Food import status	Three tier classification of high net food importer, net food importer, non-net food importer	Sourced from (20)	Reliance on imports for food supplies. Classification as a high net food importer indicates lower adaptive capacity to changes in nutrient supply

Table S5. Datasets used in analyses of nutrient flows and vulnerability framework. Datasets are ordered based on their discussion in the materials and methods section.

Dataset	Description	Use in this paper	Source	Reference
Catch	Spatially explicit wild caught marine fisheries data from the catch reconstruction. Provides tonnage of reported landings.	2015 data used for analysis of catch of nutrients.<br>1976-2015 data used to assess variability in tonnage of fish caught by foreign fishing vessels over time (vulnerability framework).	Watson & Tidd	(1)
Sea Around Us	Another commonly used global catch database. Spatially explicit wild caught marine fisheries data from the catch reconstruction. Provides tonnage of reported landings.	Compared with catch data used in this study to show results robust to selected catch dataset.	Pauly & Zeller	(2)
Trade	This database links fisheries catch data (Watson & Tidd data described above) to seafood exports and imports reported by FAO. Provides tonnage of trade flows according to importing and exporting country.	2015 data used for analysis of trade of nutrients.<br>1976-2015 data used to assess variability in tonnage of fish traded over time (vulnerability framework).	Watson et al	(5)
Flags of Convenience (FOC)	Provides information on the proportion of vessels flagged to a nation that are under FOC (averaged across 2013 and 2018).	Used to identify nutrients that may be caught by FOC vessels and thus understand the magnitude of the impact of FOCs on nutrient supplies	Petrossian	(21)
Nutrient content	Data from a trait-based Bayesian hierarchical model that predicts the concentration of 7 nutrients (protein, iron, calcium, zinc, selenium, long chain polyunsaturated omega-3 polyunsaturated fatty acids and vitamin A) per 100g raw, edible portions of fish. Models are based on a series of traits linked to the diet (feeding pathway – pelagic or benthic; trophic level), energy demand (maximum length; age at maturity; K; body shape), thermal regime (maximum depth; geographical zone) and habitat of species.	Used to estimate the nutrient concentration of all taxa reported at family, genus or species level which were caught and traded globally in 2015.	Hicks et al	(22)
Trait data	Database of fish traits	Used to source fish trait data used in nutrient content predictions.	FishBase	(23)

Level of inadequate nutrient intake for various nutrients	Country-level data on prevalence within the population of inadequate nutrient intake for four micronutrients where intake data were available: calcium, iron, zinc and vitamin A. Data is in the form of % of the population.	Used to understand the level of micronutrient deficiency risk in different countries for nutrients that are bioavailable in fish. Used to classify countries in Figure 2.	Beal et al	(15)
Coefficient of human inequality	Measure of inequalities in education, health care and income.	Used as an indicator of socio-economic status. As it was strongly correlated with GDP per capita it was not used in the vulnerability framework.	UNDP	(16)
GDP per capita	Gross domestic product divided by a nation's population size.	Used as an indicator of socio-economic status. Included in the adaptive capacity dimension of the vulnerability framework.	UNDP	(16)
Human development index	Measure of achievement in dimensions of 'long and healthy life, being knowledgeable and have a decent standard of living'	Used as an indicator of socio-economic status.	UNDP	(16)
Proportion of animal sourced protein from fish	Proportion of animal-sourced protein from marine fish that is consumed in a country.	Used as indicator of reliance on fish for food. Included in the sensitivity dimension of the vulnerability framework.	FAO	(17)
Prevalence of inadequate micronutrient intake index (PIMII)	Mean level of inadequate nutrient intake across 14 micronutrients: calcium, copper, iron, folate, magnesium, niacin, phosphorus, riboflavin, thiamin, vitamin A, vitamin B12, vitamin B6, vitamin C, and zinc. Data is in the form of % of the population.	Used to understand the level of micronutrient deficiency risk in different countries for a wide range of nutrients, not just those prevalent in fish. Included in the sensitivity dimension of the vulnerability framework.	Beal et al	(15)
Adequacy of daily energy supply	Provides the dietary energy supply (calories) as a percentage of energy required.	Indicator of hunger, but as it was strongly correlated with PIMII it was not used in the vulnerability framework.	FAO	(17)
Index of political stability and absence of violence	Measures perceptions of the likelihood that a government will be destabilized or overthrown by unconstitutional or violent means. Values from -2.97 to + 1.53	Used as an indicator of the stability of a country. Included in the adaptive capacity dimension of the vulnerability framework.	FAO	(17)
Health care expenditure as a percentage of GDP	Percentage of the GDP spent on health care.	Used as an indicator of the capacity of a nation to address nutrient deficiencies. Included in the adaptive capacity dimension of the vulnerability framework.	UNDP	(16)

Status as a food importer	Three tier classification of high net food importer, net food importer, non-net food importer.	Used as an indicator of the capacity of a nation to cope with changes in the flow of fishery-derived nutrients. Included in the adaptive capacity dimension of the vulnerability framework.	UNCTAD	(20)
Predicted change in catch due to climate change 2010 to 2050	Mean log relative change in catch 2010 to 2050 under RCP 8.5. Values from -0.44 to +0.11.	Used as an indicator of potential exposure to climate change impacts on nutrient supplies from domestic fisheries. Included in the exposure dimension of the vulnerability framework.	Blanchard et al	(14)

Dataset S1 (separate file). Percentage of animal sourced protein from fish. Nutrient concentrations of different animal-source foods.

Dataset S2 (separate file). Percentage of catch and trade flows in and out of nations only identified at low resolution (i.e. order, class or lower).

SI References and Notes

1. Watson RA & Tidd A (2018) Mapping nearly a century and a half of global marine fishing: 1869–2015. *Mar. Policy* 93:171-177.
2. Pauly D & Zeller D (2015) Catch reconstruction: concepts, methods and data sources. (University of British Columbia).
3. Roberson LA, Watson RA, & Klein CJ (2020) Over 90 endangered fish and invertebrates are caught in industrial fisheries. *Nat. Comms.* 11(1):4764.
4. de Souza EN, Boerder K, Matwin S, & Worm B (2016) Improving fishing pattern detection from satellite AIS using data mining and machine learning. *PLoS One* 11(7):e0158248.
5. Watson RA, Nichols R, Lam VWY, & Sumaila UR (2017) Global seafood trade flows and developing economies: insights from linking trade and production. *Mar. Policy* 82:41-49.
6. FAO (2020) Fishery commodities and trade. ed Division DF (Rome, Italy).
7. UNDESA (2020) Comtrade. (UN Department of Economic and Social Affairs).
8. WTO (2016) World Trade Organisation. (World Trade Organisation).
9. FAO (2020) The state of food security and nutrition in the world. (FAO, Rome, Italy), p 289.
10. FAO & WHO (2018) Strengthening nutrition action: a resource guide for countries based on the policy recommendations of the Second International Conference on Nutrition. (WHO, Geneva, Switzerland), p 96.
11. World Health Organization (2004) *Vitamin and mineral requirements in human nutrition* (World Health Organization).
12. Institute of Medicine (2005) *Dietary reference intakes for energy, carbohydrate, fiber, fat, fatty acids, cholesterol, protein, and amino acids* (National Academy Press: Washington, DC, USA).
13. FAO & WHO (2002) *Protein and amino acid requirements in human nutrition: report of a joint FAO/WHO/UNU expert consultation* (World Health Organization).
14. Blanchard JL*, et al.* (2017) Linked sustainability challenges and trade-offs among fisheries, aquaculture and agriculture. *Nat. Ecol. Evol.* 1(9):1240-1249.
15. Beal T, Massiot E, Arsenault JE, Smith MR, & Hijmans RJ (2017) Global trends in dietary micronutrient supplies and estimated prevalence of inadequate intakes. *PLoS One* 12(4):e0175554.
16. UNDP (2020) United Nations Development Programme Development Reports.
17. FAO (2019) FAOSTAT.
18. FAO (2018) The state of world fisheries and aquaculture. (FAO, Rome, Italy), p 210.
19. FAO (2019) The state of food security and nutrition in the world. (FAO, Rome, Italy), p 214.
20. UNCTAD (2020) UNCTADStat. (United Nations Conference on Trade and Development).
21. Petrossian GA, Sosnowski M, Miller D, & Rouzbahani D (2020) Flags for sale: An empirical assessment of flag of convenience desirability to foreign vessels. *Mar. Policy* 116:103937.
22. Hicks CC*, et al.* (2019) Harnessing global fisheries to tackle micronutrient deficiencies. *Nature* 574:95-98.
23. Froese R & Pauly D (2015) FishBase.
